# Ecological predictors of plant responses to sequential herbivory: a meta‐analysis

**DOI:** 10.1111/nph.70822

**Published:** 2025-12-17

**Authors:** Zoë Delamore, Julia Koricheva, Erik H. Poelman

**Affiliations:** ^1^ Laboratory of Entomology Wageningen University and Research Wageningen the Netherlands; ^2^ Department of Biological Sciences Royal Holloway University of London Egham UK

**Keywords:** induced plant resistance, meta‐analysis, multi‐herbivory, plant defence, plant life history, plant–herbivore interactions, sequential herbivory

## Abstract

- Plants evolved alongside herbivores for over 400 million years and show remarkable plasticity in responses to attack by multiple herbivores. However, it is often debated which herbivore traits predict plant responses and it is poorly understood how plant life‐history traits contribute to the variation observed in plant responses.
- We explored the role of ecological factors such as herbivore identity and plant life history by conducting a meta‐analysis of 161 studies on the effects of sequential herbivory by arthropods, nematodes and mammals. We included herbivore performance and preference as measures of induced resistance and plant growth and damage as measures of plant performance.
- We uncovered that sequential herbivory reduced herbivore performance in most cases but did not consistently affect herbivore preference. Moreover, induced resistance was particularly observed in glasshouse experiments and in experiments on cultivated plant species. Plants managed to reduce plant damage but did not reduce biomass loss effectively.
- This study highlights that plants can effectively use induced responses to defend against sequential herbivore attack regardless of herbivore identity or plant life history. To elucidate the cost of multiherbivore attack and plant adaptations to these scenarios, there is a need to examine the consequences of the interactions on plant fitness.

## Abstract 2

See also the Commentary on this article by Heinen, **250**: 699–701.

## Introduction

Most plant species experience multiple attacks by vertebrate and invertebrate herbivores. These attacks can occur simultaneously or sequentially over a plant's lifetime (Ohgushi, 2005; Poelman *et al*., 2008). Plants have evolved a wide array of defences against herbivores, including constitutively expressed chemical and morphological traits as a baseline defence, and inducible defences that are activated upon herbivore attack (Karban, 2020). Inducible defences allow plants to save metabolic costs of defences when herbivores are absent and to tailor their response to specific herbivores by adjusting the response based on the identity of the attackers and the intensity of the attack (Karban, 2020; Garcia *et al*., 2021; Fernández de Bobadilla *et al*., 2022; Ojha *et al*., 2022). Induced responses include morphological, chemical and developmental changes, such as the production of secondary metabolites that are toxic to herbivores, increased densities of spines and trichomes to prevent feeding damage and advanced/delayed development to avoid the loss of reproductive tissue (Karban & Baldwin, 1997; Karban, 2011; Poelman, 2015). As a result, herbivores may be deterred, or the performance of the attacking herbivores may be reduced (i.e. induced resistance), which should help plants to maintain their lifetime fitness (Karban & Baldwin, 1997; Karban, 2011).

Although there is compelling evidence that plants adapt to selection pressures from individual herbivore species, these adaptations are generally driven by the multitude of interactions the plant is involved in (Strauss *et al*., 2005; Mcart *et al*., 2013; Walsh, 2013; Barraclough, 2015). These adaptations shape the ecological interactions of plants, and consequently, the outcomes of multiherbivore interactions are incorporated in adaptations to optimize plant defence against multiherbivore attack (Mertens *et al*., 2021). Ecological theories around the outcome of multiherbivore interactions have identified that these interactions are often specific for the pair of herbivore species studied, their densities, the timing of interactions, feeding locations and feeding style of the herbivores involved (Ohgushi, 2005; Stam *et al*., 2014; Papadopoulou & van Dam, 2017). For example, plant family‐specific secondary metabolites, such as nicotine or glucosinolates, that are produced in response to a first herbivore may be more harmful to generalist insect herbivores than to specialist insect herbivores (Ali & Agrawal, 2012).

One hypothesis states that the feeding style and organ attacked by the first herbivore predict the resistance to a second herbivore (Stam *et al*., 2014; Papadopoulou & van Dam, 2017). When the first and second herbivores match in feeding guild, such as feeding by two different chewing caterpillar species, the induced response to one herbivore will provide cross‐resistance to the second herbivore. However, when the two herbivores differ in feeding guild, such as in a combination of a phloem feeding aphid and leaf chewing caterpillar, resistance to the second herbivore may be compromised (Thaler *et al*., 2012). This ecological hypothesis has received strong support from plant physiological constraints in regulating resistance to attack by aphids and caterpillars. The activation of signalling pathways and physiological responses of plants is often linked to the feeding guild of herbivores, with the jasmonic acid (JA) pathway being activated mostly by chewing herbivores, such as caterpillars and large grazers, and the salicylic acid (SA) pathway being activated by sap‐feeding herbivores, such as aphids (Koornneef & Pieterse, 2008; Broekgaarden *et al*., 2011; Karban, 2011). In the SA response to aphid feeding, crosstalk between phytohormonal signalling pathways that activate defence mechanisms may result in impaired JA response and may lead to increased performance of later arriving caterpillars, indicating that plants become more susceptible to future attack (i.e. induced susceptibility) (Thaler *et al*., 2012; Poelman, 2015; Vos *et al*., 2015; Aerts *et al*., 2021). However, these interactions may considerably vary in outcome because herbivores do not always activate the signalling pathways as predicted by their feeding guild, and antagonism between defence pathways does not occur in all plant species (Thaler *et al*., 2012; Ali & Agrawal, 2014; Mertens *et al*., 2021). Additionally, plant responses to herbivory are regulated by more than just the JA and SA pathways, and phytohormone levels are affected by plant age, abiotic conditions and other simultaneous stresses (Aerts *et al*., 2021).

One may also expect considerable variation in plant responses to interactions with multiple herbivores. From the plant's perspective, defence strategies to sequential attack may depend on plant ontogeny, the location of the tissue that gets attacked or plant life history (Johnson *et al*., 2012; Moreira *et al*., 2018; Garcia *et al*., 2021). As defence is a way to preserve fitness, it is especially important for annual plants to minimize the impact of herbivores, whereas perennial plants may have multiple years to ensure the production of offspring. Moreover, the number and diversity of herbivore species in the community associated with a plant species, as well as the predictability of herbivore arrival on individual plants, may determine how plants deal with sequential herbivore attack (Mertens *et al*., 2021; Fernández de Bobadilla *et al*., 2022). Additionally, plants may change their defence strategy during development, with investing more in the protection of reproductive tissue than leaves (McKey, 1974; Barton & Koricheva, 2010). However, as most of our evidence of how plants respond to sequential herbivory is based on measurements of herbivore performance instead of the reduction of fitness loss due to herbivory, it is poorly understood how plants are affected in their growth and fitness by being attacked by multiple herbivores over their lifetime (Poelman, 2015; Erb, 2018).

Even though herbivore identity and plant life history are considered important factors in regulating plant responses to herbivory, a unified view on how these factors determine the outcome of sequential herbivory is missing. Moreover, it is unclear how the different outcomes that are used as proxies for plant defence contribute to our understanding of plant responses to sequential herbivory. While the focus has often been on herbivore performance, finding the key to understanding plant defence may need a more thorough understanding of plant performance (Poelman, 2015). To understand plant defence strategies in complex environments with multiple herbivores, it is essential to find good predictors for plant responses to sequential herbivory. Therefore, this meta‐analysis aims to understand plant defence strategies to sequential herbivore attack by focussing on herbivore performance and preference as measures of plant resistance and plant growth, feeding damage and fitness as measures of plant performance. Specifically, we ask how plant life history, herbivore identity and feeding behaviour alter plant responses to sequential attack by two herbivore species compared to that after attack by a single herbivore to an undamaged plant. We test the hypothesis that (1) sequential attack by herbivores with similar life‐history traits (e.g. feeding guild, feeding location) leads to induced resistance and increased plant performance, whereas combinations of herbivores with different traits may lead to induced susceptibility and reduced plant performance, (2) annual plants show stronger induced responses than perennial plants, and (3) experimental conditions modulate induced responses.

## Materials and Methods

### Literature search and data extraction

Literature search was conducted in June 2023 in the Web of Science Core Collection and Scopus, using the following search strings:

Search string Web of Science core collections: Search string: TS=(((belowground OR root*) AND herbivor* AND (attack OR feeding OR damage)) OR ((multi* OR sequen* OR dual OR subsequent OR consecutive OR order) AND (insect* OR arthropod* OR mite*) AND (plant* OR tree* OR crop*) AND herbivor* AND (induction OR induce* OR vaccination)) OR (plant* AND (browsing OR mammal* OR ungulate*) AND (insect* OR arthropod*) AND (induce* OR induction))).

Search string Scopus: TITLE‐ABS‐KEY (((belowground OR root*) AND herbivor* AND (attack OR feeding OR damage)) OR ((multi* OR sequen* OR dual OR subsequent OR consecutive OR order) AND (insect* OR arthropod* OR mite*) AND (plant* OR tree* OR crop*) AND herbivor* AND (induction OR induce* OR vaccination)) OR (plant* AND (browsing OR mammal* OR ungulate*) AND (insect* OR arthropod*) AND (induce* OR induction))).

Additionally, a forward and backward search was performed using Web of Science using both well‐cited and recently published relevant papers in the field (Hoffmann & Schausberger, 2012; Johnson *et al*., 2012; Thaler *et al*., 2012; Wondafrash *et al*., 2013; Stam *et al*., 2014; Moreira *et al*., 2018; Garcia *et al*., 2021; Fernández de Bobadilla *et al*., 2022). All search results were pooled and deduplicated based on the Digital Object Identifier (DOI). This resulted in 5457 unique papers published between 1985 and 2023 (Supporting Information Fig. S1). We screened titles and abstracts using the ‘abstract_screener’ function from the R package metagear (Lajeunesse, 2016). The main criteria for study inclusion in abstract screening and the following full‐text assessment were as follows:

1. Herbivores fed on a terrestrial plant species.
2. The treatment included sequential attack by two groups of herbivores on the same plant.
3. The control included a single herbivore attack by the second herbivore of the sequence with the same herbivore density on a previously undamaged plant.
4. The response types reported included performance or preference of the second herbivore, or plant performance parameters.

After abstract screening, the full text of the remaining papers was assessed. Experiments or parts of experiments were excluded if the criteria above were not met or if one of the following exclusion criteria occurred: Herbivory was simulated using mechanical damage and/or phytohormone application; additional treatments were imposed besides herbivory (e.g. arbuscular mycorrhizal fungi, extreme abiotic conditions and pesticide treatments); multigenerational or epigenetic studies that measure the outcome on a plant other than that herbivores fed on; outcomes that do not directly measure resistance or performance (e.g. gene expression); and plant or herbivore species used in the experiment were not mentioned.

Data were extracted from text, tables and figures. The free/open‐source software WebPlotDigitizer v.4.6 (https://apps.automeris.io/wpd/) was used to extract data from figures. Axes and datapoints were manually selected. If raw data were available, these were used to extract relevant data.

Information about the plant and herbivore species, as well as experimental design, was recorded from each study. For plants, the plant species, duration of life cycle (annual/perennial) and growth form (herbaceous/woody), and whether the species was wild or cultivated were obtained. For herbivores, the species, feeding guild, diet breadth and feeding location (above‐ or belowground) were included. The experimental design details included the duration of each experimental step, the experimental setting (e.g. field or glasshouse) and the type of response measured (i.e. herbivore performance, herbivore preference and plant performance). All durations were converted into number of days.

### Calculation of effect sizes

For each experimental outcome, we calculated the standardized mean difference (Hedges' *g*) using the metafor package in R (Hedges, 1981; Viechtbauer, 2010), where:

$$g=J\frac{{\bar{x}}_{\text{treatment}}-{\bar{x}}_{\text{control}}}{\sqrt{\sigma }}$$

with pooled standard deviation:

$$\sigma =\frac{\left(n_{\text{treatment}}-1\right)\sigma_{\text{treatment}}^{2}+\left(n_{\text{control}}-1\right)\sigma_{\text{control}}^{2}}{n+n-2}$$

where x refers to the plant or herbivores response, sample size n, and

$$J=1-\frac{3}{4\left(n_{\text{treatment}}-n_{\text{control}}-2\right)-1}$$

Plant and herbivore responses were divided into three categories: herbivore performance, herbivore preference and plant performance. First, herbivore performance was classified as either survival, growth, fecundity or development time of an herbivore on a plant induced by a prior herbivore (treatment) compared to that on an undamaged plant (control). For studies with development time as outcome, the sign of *g* was reversed to give the effect size a consistent meaning. A negative *g* indicates lower herbivore performance on induced plants compared with the control, that is induced resistance. Second, herbivore preference was measured as the feeding or oviposition choice of an herbivore between an induced and undamaged plant. A negative *g* indicates preference by the second herbivore for control plants, while a positive *g* indicates preference for induced plants. Last, plant performance was assessed as plant biomass gain, the area of tissue damage or reproductive output after experiencing sequential or single herbivore attack. For studies with damage as outcome, the sign of *g* was reversed to give the effect size a consistent meaning. A negative *g* indicates lower plant performance after sequential herbivore attack compared with single herbivore attack.

### Meta‐analysis

We first calculated an overall mean effect and confidence interval (CI) for each response type using random effects models to assess whether there was an overall effect of herbivore induction on plant performance or subsequent herbivore performance or preference. The effect was considered statistically significant if the CI did not overlap with zero. Then, we constructed multilevel metaregression models for each of the moderators and included *Q*‐tests to estimate moderator effects. All models were constructed using the ‘rma.mv’ function in the metafor package in R (Viechtbauer, 2010). Model estimates were obtained from the ‘mod‐results’ function of the R package orchard (Nakagawa *et al*., 2023), and results were visualized using R packages orchard and ggplot2 (Wickham, 2016). We accounted for nonindependence among effect sizes from the same study by including the study ID as a random effect to account for multiple experiments within a paper. In addition, we included a treatment ID nested within study ID as a random effect to account for multiple treatments that were compared with the same control within an experiment.

The moderators tested for each response type were divided into herbivore traits, plant traits and experimental design. Herbivore traits included feeding guild, diet breadth and location of attack and were assigned based on traits mentioned in the original papers. Plant traits included duration of the life cycle, growth form and whether the species was wild or cultivated. Experimental design included whether the experiment was performed in the field or glasshouse; whether the species of the subsequent herbivore was the same as the first herbivore; whether the first herbivore was removed before adding the subsequent herbivore; and the duration of each round of herbivore feeding. Any confounding between moderators was described in the Results section.

### Sensitivity analysis

#### Correcting for phylogeny and overrepresentation of species

To assess the effect of phylogenetic relatedness on the results, all models were repeated with plant or herbivore phylogenies as an additional random factor. As including multiple phylogenies in one model interfered with parameter fitting, three separate models were created for either plant, inducing herbivore and subsequent herbivore phylogeny. Phylogenies were constructed by pruning existing megatrees using the u.phylomaker package (Jin & Qian, 2023). Phylogenies were converted into variance–covariance matrices and included as random factors in all models mentioned previously (Cinar *et al*., 2022; Nakagawa *et al*., 2023). In case a species, or closely related species as a substitute, was not included in the megatree, it was excluded from the sensitivity analysis.

Separately, to examine the effect of often‐studied species, we reran analyses on the response types nine times, excluding either one plant or one herbivore species (either inducing herbivore or subsequent herbivore). The three most‐studied species in each group were chosen for exclusion. For plants, the excluded species were *Brassica oleracea* L. (13.5% of observations), *Brassica nigra* L. (11.8%) and *Solanum lycopersicum* L. (10.8%). For inducing herbivores, the excluded species were *Brevicoryne brassicae* L. (5.9%), *Plutella xylostella* L. (5.5%) and *Meloidogyne incognita* Chitwood (5.4%). For subsequent herbivores, the excluded species were *Tetranychus urticae* Koch (7.4%), *Myzus persicae* Sulzer (5.9%) and *P. xylostella* (5.9%).

#### Validation with additional data

A subset of studies (*n* = 31) that examined herbivore survival or preference reported individual herbivore survival or choice instead of a mean and SD. Therefore, instead of Hedges' *g*, we calculated a log odds ratio (LOR) as an alternative effect size using the metafor package in R (Viechtbauer, 2010), where:

$$\mathrm{LOR}=\log\left(\frac{\left(p1/1-p1\right)}{\left(p2/1-p2\right)}\right)$$

with

$$p1=\frac{p_{\text{treatment}}}{n_{\text{treatment}}}$$

and

$$p2=\frac{p_{\text{control}}}{n_{\text{control}}}$$

where *p* refers to the number of individuals surviving on or choosing the control plants and n refers to the sample size per group. A negative LOR indicates preference for or higher survival on the control plants, while a positive LOR indicates preference for or higher survival on the induced plants. Meta‐analysis of odds ratios was then performed as described previously.

### Publication bias analysis

We tested for publication bias using several methods for each of the main response types (herbivore performance, herbivore preference and plant performance). First, we examined changes in effect sizes with publication year by calculating the cumulative effect sizes for each study using the ‘cumul’ function of the metafor package. Next, we produced funnel plots and tested their asymmetry using Egger's regression test (‘regtest’ function in the metafor package).

## Results

**Fig. 1:**
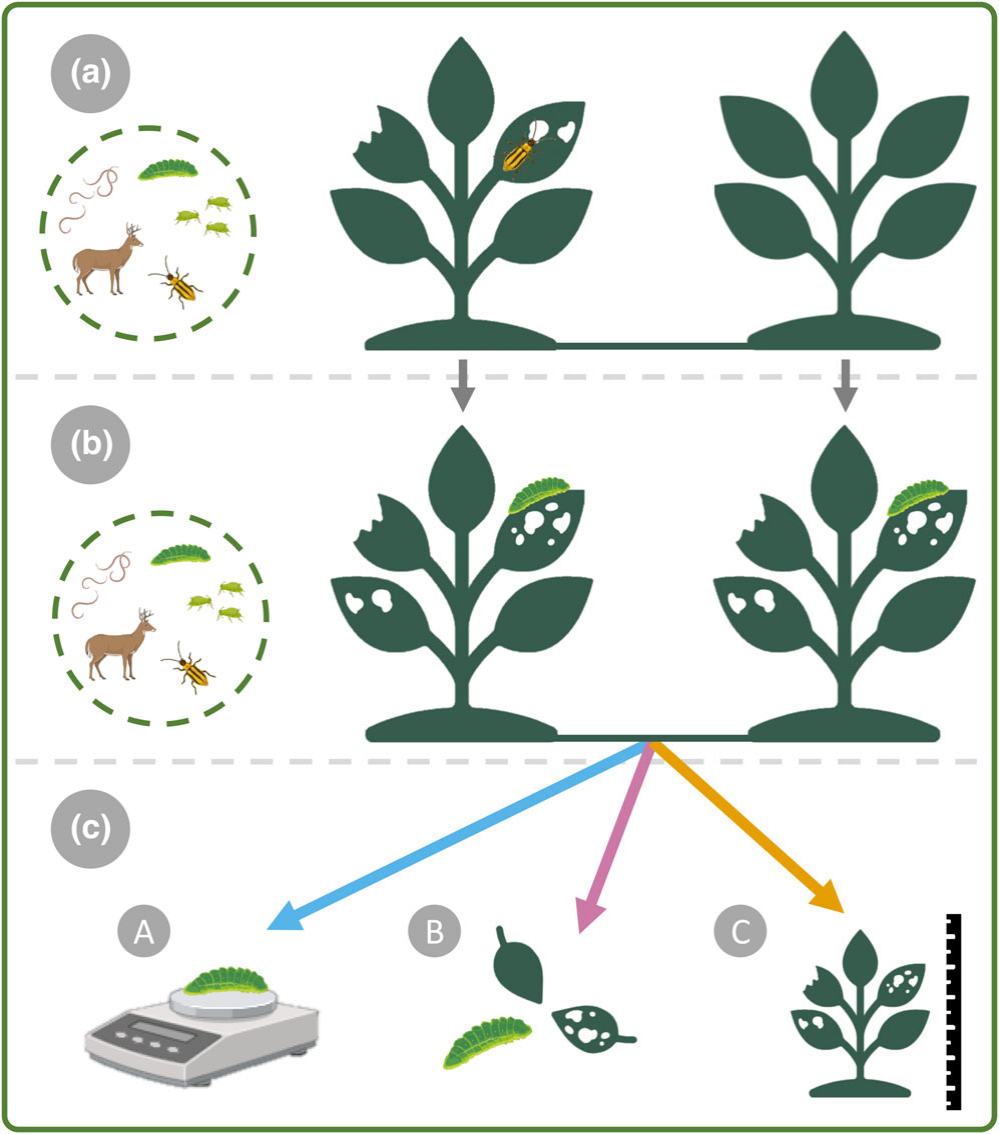
Summary of the experimental design used in sequential herbivore attack studies included in this meta‐analysis. (a) Induction of plant responses by one herbivore species (either an arthropod, nematode or mammal, depicted in the dashed circle), with an undamaged plant as a control. (b) Subsequent herbivore attack on both the induced and previously undamaged plants by one herbivore species. (c) Assessment of plant responses by measuring herbivore performance (A), herbivore preference (B) or plant performance (C). This figure was created in BioRender (https://BioRender.com/qym9qha).

The literature search yielded 161 suitable papers from which 1505 effect sizes were extracted (Fig. S1; Notes S1). Each effect size (Hedges' *g*) represents the difference between the plant or herbivore response after sequential attack by two herbivores compared with the response after a single herbivore attack (Fig. 1). A wide variety of species were used in the studies, including 67 plant species from 29 plant families and 146 herbivore species, including insects, mites, nematodes and mammals (Fig. S2). The most‐studied plant species were *B. oleracea*, *B. nigra* and *S. lycopersicum*, with over 100 effect sizes each. Overall, the list of studied plant species was dominated by annual crops, followed by wild annual herbs. Among herbivores, chewing insects were best represented, followed by sap‐feeding insects. The most‐studied species were the aphid *B. brassicae*, nematode *M. incognita* and caterpillars of *P. xylostella* as inducing herbivores, and the aphid *M. persicae*, *P. xylostella* and the spider mite *T. urticae* as subsequent herbivores. Studies on mammalian herbivores that fit the inclusion criteria were scarce, with only 15 effect sizes for mammals as the inducing herbivore and none as the subsequent herbivore. Most studies explored aboveground herbivores (1036 effect sizes), while solely belowground interactions were only included in 22 effect sizes, and below‐/aboveground and above‐/belowground represented by 232 and 161 effect sizes, respectively.

### Induced plant responses decrease herbivore performance but also reduce plant growth

**Fig. 2:**
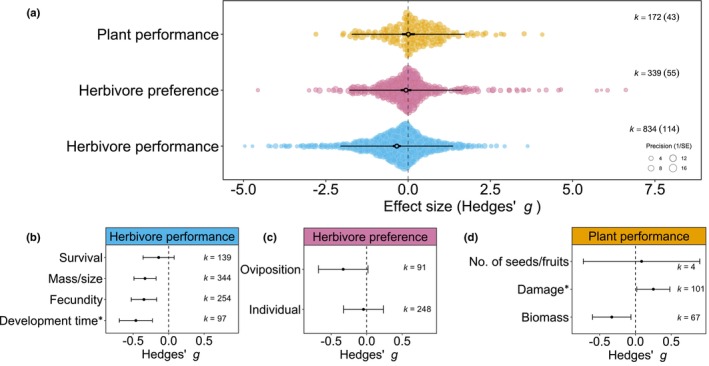
Main effects of sequential herbivory on herbivore performance, herbivore preference and plant performance. Orchard plots showing the mean effect sizes (Hedges' *g*) for effects of sequential herbivory on plant performance, herbivore preference and herbivore performance (a). Bold lines show 95% confidence interval (CI); thin lines the 95% prediction interval; coloured points the individual effect sizes. *k* = the number of effect sizes included, with the number of studies between brackets. Influence of outcomes measured (Hedges' *g* ± 95% CI) on herbivore performance (b), measures of herbivore preference (c) and measures of plant performance (d). For response variables indicated with an asterisk, the sign of the effect has been reversed to ensure consistent interpretation of measured responses (i.e. positive effects indicate higher herbivore or plant performance following sequential herbivory). *k* = the number of effect sizes included.

As plant responses to sequential herbivory were measured using various outcomes, the experimentally measured responses were divided into three categories – herbivore performance, herbivore preference and plant performance – and analysed separately. Each of the outcomes showed considerable variation, with each of the outcomes showing cases of increased resistance and increased susceptibility after sequential herbivory (Fig. 2a). This variation could partly be attributed to specific outcomes measured. First, herbivore performance responses included differences in survival, growth, fecundity or development time of an herbivore on an induced plant compared with an undamaged plant. The mean negative effect size for herbivore performance indicated that herbivores performed better on undamaged than induced plants, suggesting higher resistance of induced plants (Hedges' *g* = −0.326, 95% CI = [−0.453, −0.200], *k* = 834, *I* ^2^ = 87.9%). Sequential herbivory significantly reduced the mass and fecundity of second herbivores and increased their development time compared with the performance of the same herbivore on undamaged plants but did not significantly affect survival (Fig. 2b). Moreover, the magnitude of the effect did not differ significantly between the herbivore performance outcomes (Qm = 5.7, df = 3, *P* = 0.127).

Second, when responses were measured in terms of herbivore preference (i.e. feeding or oviposition choice of an herbivore between an induced and undamaged plant), herbivores had no overall preference for undamaged or induced plants (Hedges' *g* = −0.142, 95% CI = [−0.403, 0.119], *k* = 339, *I* ^2^ = 95.1%). In choice experiments, subsequent insect herbivores tended to lay eggs on undamaged plants compared with induced plants, while the effect size for individual feeding choice was near zero, suggesting no preference (Fig. 2c). Moreover, the two outcomes did not differ significantly (Qm = 2.5, df = 1, *P* = 0.114).

Third, plant performance was similar after receiving single or sequential herbivory (Hedges' *g* = −0.013, 95% CI = [−0.221, 0.196], *k* = 172, *I* ^2^ = 85.5%). The two most common indicators used for plant performance – damage and biomass – showed that plants are more resistant to the subsequent herbivore by preventing tissue loss but do not mitigate biomass loss completely. Neither damage nor biomass differed from the closest indicator for plant fitness, the number of seeds and fruits (Qm = 11.4, df = 2, *P* = 0.003; Fig. 2d).

### Plant resistance is maintained or increased with varying herbivore identity

**Fig. 3:**
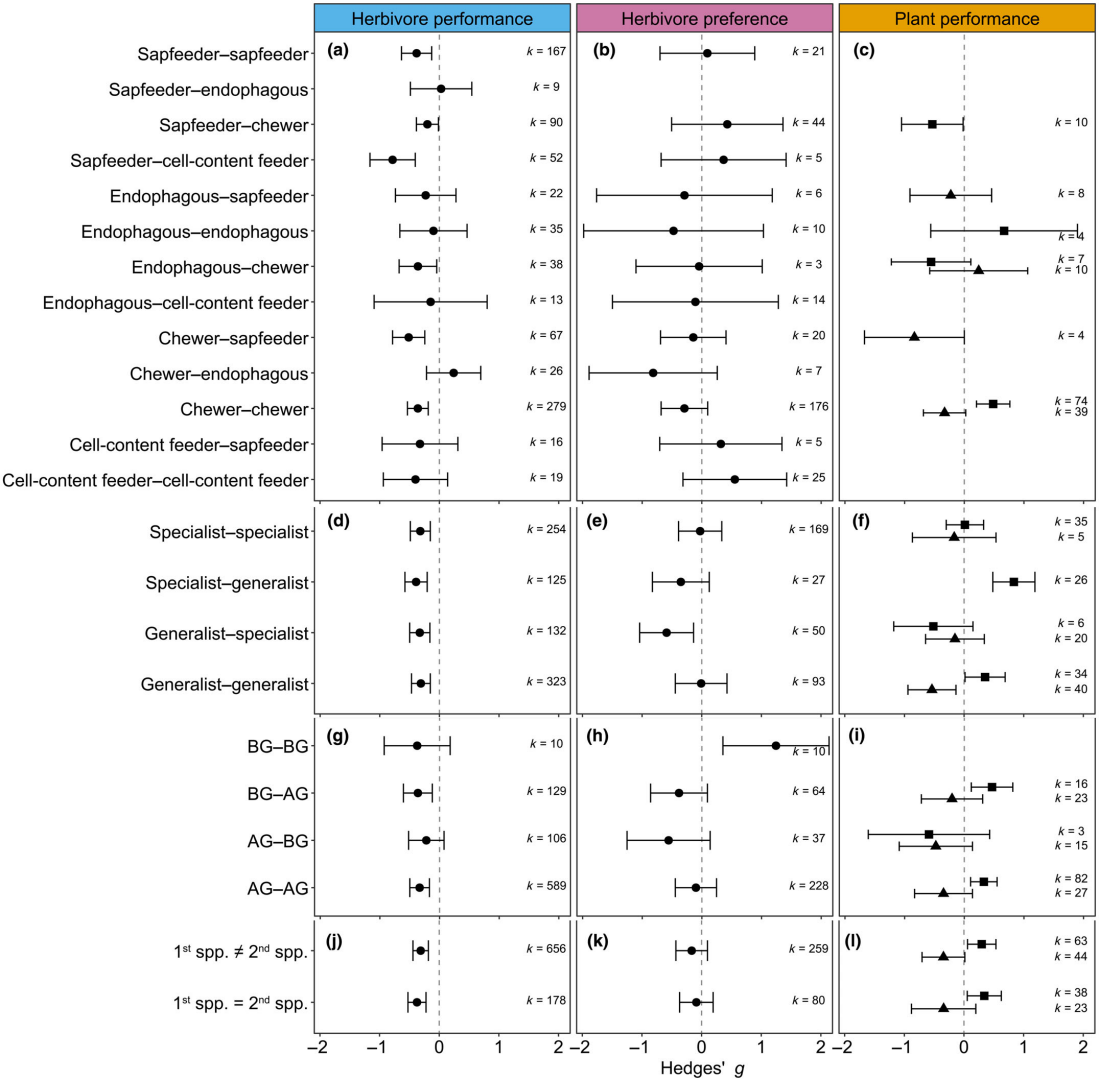
Influence of herbivore traits on the effects of sequential herbivory on herbivore performance, herbivore preference and plant performance (Hedges' *g* ± 95% confidence interval): feeding guild (a–c), diet breadth (d–f), feeding location (BG, belowground; AG, aboveground; g–i) and species identity (j–l). The first traits in the sequence refer to the inducing herbivore, the second trait to the subsequent herbivore. Plant performance is divided into biomass (mean effect size indicated with triangles) and damage (mean effect size indicated with squares). *k* = the number of effect sizes included. Missing bars indicate no data were available.

Herbivore identity was defined by several traits, including feeding guild, phylum, diet breadth, feeding location and whether the inducing and subsequent herbivores were the same or different species. Overall, the effects of these traits varied with the measured response type, in which herbivore performance more often differed between sequential and single herbivore attack and showed less variation than herbivore preference and plant performance (Fig. 3). Groups with high sample sizes generally showed significant decreases in herbivore performance of the subsequent herbivore than single herbivore attack, while the large variability in effect size in groups with low sample sizes resulted in nonsignificant differences between sequential and single herbivory.

Our results show that induced resistance generally occurs in response to sequential herbivory by both the same and different feeding guilds. Several herbivore feeding guilds performed worse on induced than undamaged plants, and there were significant differences between feeding guild combinations (Qm = 29.714, df = 13, *P* = 0.005). Sequential attack by herbivores of the same feeding guild led to either induced resistance or neutral effects. However, contrary to our expectations, sequential attack by herbivores of different feeding guilds also led to induced resistance or neutral effects (Fig. 3a). For example, the performance of chewing herbivores was significantly reduced after feeding by sap feeders. However, the magnitude of induced resistance was lower than after sequential feeding by two chewing herbivores. Moreover, sequential feeding by chewers led to less plant damage, while having a chewer after sap feeders led to more damage than single herbivory by chewers (Fig. 3c), and plant damage differed significantly between combinations of feeding guilds (Qm = 30.383, df = 7, *P* < 0.001). There were no significant effects of feeding guild on herbivore preference (Qm = 9.645, df = 13, *P* = 0.723) or plant biomass (Qm = 12.408, df = 6, *P* = 0.053; Fig. 3c). These results indicate that there may be a cost for plants to switch defence strategies between feeding guilds but that does not lead to induced susceptibility.

The combinations of attack by herbivores with varying diet breadth led to differences in herbivore preference (Qm = 9.874, df = 3, *P* = 0.020; Fig. 3e) and plant damage (Qm = 17.241, df = 3, *P* = 0.001; Fig. 3f), but not herbivore performance (Qm = 1.029, df = 3, *P* = 0.794; Fig. 3d) or plant biomass (Qm = 2.813, df = 3, *P* = 0.421). Herbivores generally performed worse on induced plants, regardless of the diet breadth combinations. Specialist herbivores preferred undamaged plants over plants induced by generalist herbivores but showed no preference when choosing between undamaged plants and plants induced by specialist herbivores. Plant damage by generalists was lower after induction by generalist or specialist herbivores than on undamaged plants, while specialists caused similar damage levels on undamaged plants and plants induced by either generalists or specialists. Together, these results suggest that induced plants can limit damage by generalist herbivores but not specialist herbivores, and plant‐induced defences do not alter generalist and specialist herbivore performance differently compared with their respective controls.

The feeding location of herbivores was defined as either above‐ or belowground. Aboveground herbivores performed better on undamaged than on induced plants, while belowground herbivores performed similar on undamaged and induced plants, although the mean effects were not significantly different among feeding location combinations (Qm = 0.768, df = 3, *P* = 0.857; Fig. 3g). Contrastingly, herbivore preference significantly differed among feeding location combinations (Qm = 19.250, df = 3, *P* < 0.001). Belowground herbivores preferred plants induced by other belowground herbivores compared with undamaged plants, but not plants induced by aboveground herbivores (Fig. 3h). Additionally, plant damage by aboveground herbivores was lower on induced than undamaged plants but did not differ significantly from belowground herbivores (Qm = 3.807, df = 2, *P* = 0.149; Fig. 3i). Plant biomass was similar for all feeding combinations and did not significantly differ from zero (Qm = 0.808, df = 3, *P* = 0.848).

A special case of sequential herbivory is when the inducing and subsequent herbivore are the same species. As expected, subsequent herbivores showed slightly lower performance when the inducer was of the same species compared with situations in which the two herbivores were different, but the difference was not statistically significant (Qm = 1.212, df = 1, *P* = 0.271; Fig. 3j). Also, responses in terms of herbivore preference (Qm = 1.112, df = 1, *P* = 0.292; Fig. 3k), plant damage (Qm = 0.078, df = 1, *P* = 0.779), and plant biomass (Qm = 0.001, df = 1, *P* = 0.993) did not differ between sequential herbivory from conspecifics and heterospecifics (Fig. 3l).

### Plant life history is a poor predictor for strength of induced plant resistance

**Fig. 4:**
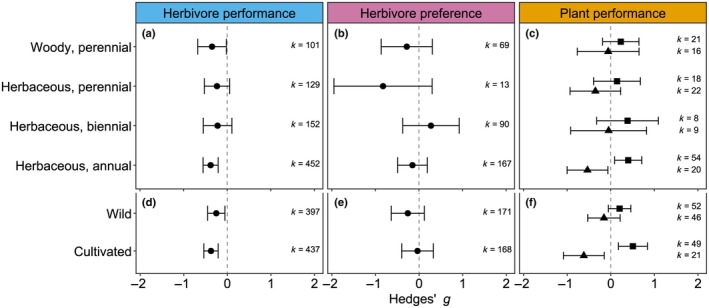
Influence of host plant life history and cultivation status on the effects of sequential herbivory (Hedges' *g* ± 95% confidence interval) on herbivore performance (a, d), herbivore preference (b, e) and plant performance (c, f). Plant performance is divided into biomass (mean effect size indicated with triangles) and damage (mean effect size indicated with squares). *k* = the number of effect sizes included.

Herbivore performance and preference were similar on undamaged and induced plants across different plant life histories (performance: Qm = 1.183, df = 3, *P* = 0.757; Fig. 4a; preference: Qm =3.220, df = 3, *P* = 0.459; Fig. 4b). The mean effect size of herbivore performance was always negative, suggesting that herbivores perform better on undamaged plants than on induced plants, but the herbivore performance on induced plants was statistically different from that on undamaged plants only for annual herbaceous plants (Fig. 4a). Plant performance of annual herbs showed the same pattern as the main responses, in which induced plants can prevent damage by the subsequent herbivore but produce less biomass (Fig. 4c). The majority of the annual herbs were cultivated species, confounding plant life history with cultivation status. However, whether plant species were cultivated or wild species did not significantly alter responses in terms of herbivore performance (Qm = 0.855, df = 1, *P* = 0.355; Fig. 4d), herbivore preference (Qm = 0.694, df = 1, *P* = 0.405; Fig. 4e) or plant performance (Qm = 0.006, df = 1, *P* = 0.938; Fig. 4f).

### Effects of experimental design

**Fig. 5:**
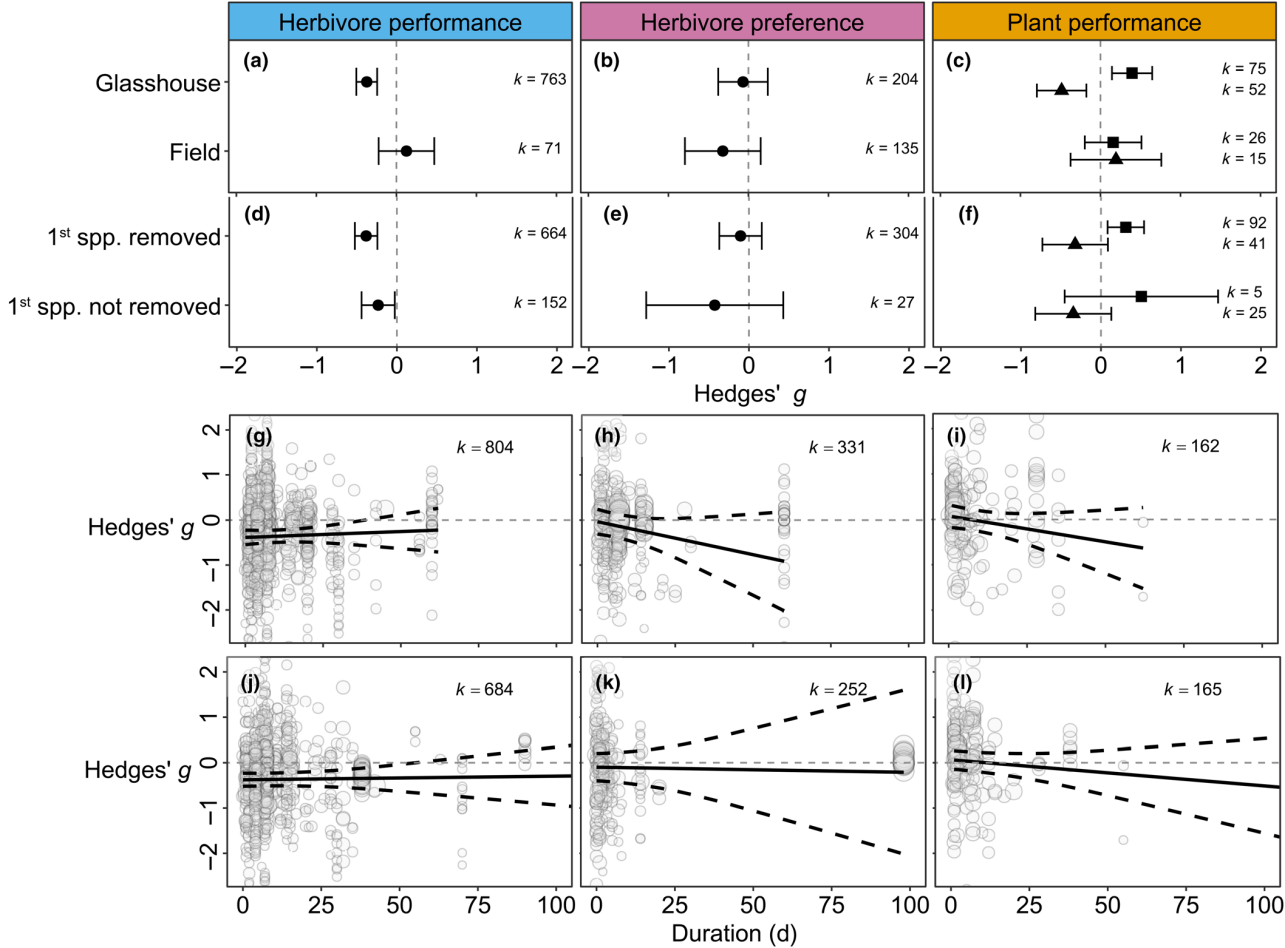
Influence of the experimental design (Hedges' *g* ± 95% CI) on the effects of sequential herbivory on herbivore performance, herbivore preference and plant performance for glasshouse vs field experiments (a–c) and removal of inducing herbivore (d–f). Plant performance is divided into biomass (mean effect size indicated with triangles) and damage (mean effect size indicated with squares). Brackets indicate significant differences between groups. Influence of the duration of inducing herbivore attack (g–i) and duration of subsequent herbivore attack (j–l) on herbivore performance, herbivore preference and plant performance. Solid black lines represent the mean effect (Hedges' *g*), and dashed black lines represent the 95% CI around the mean. *k* = the number of effect sizes included.

Induced resistance, that is lower subsequent herbivore performance, was observed in glasshouse studies but not in field experiments (Qm = 7.486, df = 1, *P* = 0.006; Fig. 5a), suggesting that the strength of induced resistance might be overestimated in glasshouse experiments compared with natural situations in which plants may be challenged and responding to a wide range of biotic and abiotic conditions. Additionally, the effect of induction on plant biomass was significantly larger in glasshouse than in field experiments (Qm = 4.240, df = 1, *P* = 0.039; Fig. 5c), while the effects on herbivore preference (Qm = 0.782, df = 1, *P* = 0.376; Fig. 5b) and plant damage (Qm = 1.143, df = 1, *P* = 0.285; Fig. 5c) were consistent among glasshouse and field experiments.

In the majority of experiments, the inducing herbivore was removed before the subsequent herbivore was placed, but in some studies, inducers remained on the plant throughout the experiment. Although it was hypothesized that this may change the response to the subsequent herbivore, effect sizes were similar for herbivore performance (Qm = 1.875, df = 1, *P* = 0.171; Fig. 5d), herbivore preference (Qm = 0.496, df = 1, *P* = 0.481; Fig. 5e) and plant performance (biomass: Qm = 0.006, df = 1, *P* = 0.941; damage: Qm = 0.149, df = 1, *P* = 0.699; Fig. 5f) in both types of experiments. Additionally, no significant effects were found for the duration of attack from the inducing or subsequent herbivore on herbivore performance (1st attack: Qm =0.313, df = 1, *P* = 0.576; Fig. 5g; 2nd attack: Qm = 0.048, df = 1, *P* = 0.826; Fig. 5j), herbivore preference (1st attack: Qm = 1.986, df = 1, *P* = 0.159; Fig. 5h; 2nd attack: Qm = 0.013, df = 1, *P* = 0.908; Fig. 5k) or plant performance (1st attack: Qm = 1.866, df = 1, *P* = 0.172; Fig. 5i; 2nd attack: Qm = 0.992, df = 1, *P* = 0.319; Fig. 5l). However, increased durations of the first herbivore seem to increase herbivore preference for undamaged plants (Fig. 5h) and decrease plant performance (Fig. 5i).

### Publication bias

The magnitude of effect sizes varied with publication year. The mean cumulative effect size for herbivore performance showed fluctuation in early years but stabilized at a Hedges' *g* between −0.3 and −0.5 since 2011 (Fig. S3A). The cumulative effect size for herbivore preference has increased over time from negative effect sizes to a current cumulative effect size close to zero (Fig. S3B). For plant performance, the mean effect size remained stable over the last decades (Fig. S3C). Funnel plots revealed asymmetric distributions for herbivore performance and preference, with a negative skew for herbivore performance (*Z*_834_ = −6.465, *P* < 0.001; Fig. S4A), a positive skew for herbivore preference (*Z*_339_ = 4.506, *P* < 0.001; Fig. S4B). Meanwhile, plant performance had a symmetrical distribution (*Z*_172_ = 1.361, *P* = 0.174; Fig. S4C). Egger's regression did not show a relationship between the inverse of variance and effect size (slope = 0.051 ± 0.041, *F*_1,1343_ = 1.533, *P* = 0.216), suggesting that precision was similar across different magnitudes of effect sizes.

### Sensitivity analysis

A sensitivity analysis was performed to assess the effects of phylogenetic relationships and overrepresented species on the results. The inclusion of plant and herbivore phylogenies resulted in similar statistical outcomes when testing the effects of moderators (Table S1) and had minor effects on means of model estimates. CIs widened when accounting for phylogeny, which meant that some differences in response between undamaged and induced plants became nonsignificant. This could be attributed to some species being overrepresented, so correcting for phylogenetic relatedness reduced the weight of these species. This suggests that some effects found in the main analysis may be confounded by species‐specific effects, rather than representing a whole group of species.

To assess how the most‐studied species contributed to the phylogenetic effects, the three most‐studied species from each group (plants, inducing herbivores and subsequent herbivores) were excluded one at a time, and models constructed for the main effects of the different responses measured were reconstructed. In only one instance, the mean estimate was significantly different from the main analysis. Removing *P. xylostella* as an inducing herbivore from the data led to a lower Hedges' *g* than the main analysis and widened the CI to include zero, which would mean that the effect on damage being positive is largely driven by *P. xylostella* (Table S2). Additionally, two minor changes in CIs occurred, but with similar model estimates (Table S2).

#### Validation with additional data

The subset of data for which LORs were calculated showed similar outcomes to the main results. Survival was significantly lower on induced plants than on the clean plants (LOR = −0.80, 95% CI = [−1.34, −0.27], *I* ^2^ = 50.4%; Fig. S5A). Although the effect was not statistically significant in the main analysis, the direction of the effect was the same and in line with other herbivore performance results. Also, herbivore preference studies had similar results. Individual feeding choice did not seem to differ between undamaged and induced plants, while herbivores preferred to oviposit on undamaged plants. While the result was not statistically significant in the main analysis, here feeding and oviposition choice differed significantly (Qm = 5.702, df = 1, *P* = 0.017; Fig. S5B).

## Discussion

To understand how plants can defend against sequential herbivore attack, we performed a meta‐analysis in which we tested the effect of herbivore and plant life history on induced plant responses. Overall, induced plant responses were effective in increasing or maintaining resistance against subsequent attackers. Contrary to the hypotheses that attack by two herbivores with different traits may cause induced susceptibility, we found that also in these cases, the majority of studies reported induced resistance or similar resistance in single and sequential attack. Nevertheless, in interactions between sap feeders and chewers, the magnitude of induced resistance may be lower than in interactions between two chewers. Moreover, induced resistance was stronger in glasshouse studies than in field studies. The reduced performance of herbivores often did not correspond with the response in herbivore preference and could generally not be extrapolated to significant reduction of plant damage or mitigation of herbivory on plant performance. Only in the case of sequential attack by belowground herbivores did induced plant responses lead to increased attraction by other belowground herbivores, but this was not reflected in effects on herbivore performance. Data on plant fitness were mostly lacking. Across plant life‐history strategies, induced responses to the first herbivore led to reduced performance of a second herbivore. Only annual plants showed significant reductions in plant damage while decreasing the performance of the subsequent herbivores. These effects were consistent across glasshouse and field studies. However, most annual plants were cultivated species, and wild species did not show effects of sequential herbivory on plant performance. In general, sequential herbivory reduced subsequent herbivore performance and plant damage but did not impact herbivore preference.

Although our effect sizes in herbivore performance were small to moderate (Sawilowsky, 2009; Cohen, 2013), plant responses to sequential attack consistently reduced herbivore performance or resulted in resistance levels similar to a single herbivore attack. Induced resistance was observed with combinations of attack by both the same feeding guild and different feeding guilds. Moreover, the level of induced resistance was similar after sequential attack by the same species vs different species. Together, these findings indicate that plants across all included plant families are well adapted to deal with sequential attack by herbivores of different identities and feeding styles. This also means that even though plants respond with different signal transduction pathways to sap feeders and chewers, and despite the antagonistic crosstalk between SA and JA, this typically does not result in induced susceptibility (Favery *et al*., 2020). Our study highlights that the magnitude of resistance to dual herbivore attack is lower in a combination of sap feeders and chewers than dual chewer attack. This suggests that at least in some situations, a response to sap feeders reduces effectiveness in resistance to chewers. This may be caused by antagonistic crosstalk between SA and JA (Thaler *et al*., 2012; Moreira *et al*., 2018) or could be explained by other physiological consequences of a response to sap‐feeding herbivores such as reduced nutrient quality of plants to the second herbivore (Denno *et al*., 2000; Canepuccia *et al*., 2020). While most feeding guild combinations showed induced resistance, CIs generally overlapped with zero. This suggests that across feeding guilds, individual species differ in the induced response that they initiate. For example, the sap‐feeding aphid *B. brassicae* (Stam *et al*., 2014) and chewing caterpillars of the diamondback moth *P. xylostella* induce responses that are both characterized by a profile of SA, JA and additional phytohormone pathways (Ehlting *et al*., 2008). Overall, our meta‐analysis suggests that plants induce responses that acquire resistance to the majority of their attackers (Karban, 2020; Mertens *et al*., 2021). This could mean that the first herbivore, given that it does not kill the plant, is most important as a cue for future herbivory and is used by plants to anticipate future attack (Mertens *et al*., 2021). Moreover, early herbivory on vegetative tissue may provide enough time for plants to optimize their defences to protect valuable reproductive tissue later in the season (Barton & Koricheva, 2010; McCall & Fordyce, 2010).

In our meta‐analysis, plants showed the same magnitude of induced resistance against specialist and generalist herbivores, regardless of the host plant specialization of the inducing herbivore. These findings do not match the hypothesis that generalist herbivores may be more negatively affected in their performance by induced plant responses than specialist herbivores. A likely explanation is the difference in effect of constitutive resistance levels against herbivores with different host plant specializations, which could result in overall lower performance of generalist herbivores on both undamaged and induced plants (Ali & Agrawal, 2012). Alternatively, the categorization of herbivores into generalists and specialists may be too crude to distinguish between plant defence strategies against herbivores with different diet breadths (Ali & Agrawal, 2012). In line with this, the level of food plant specialization did not explain the large variation in preference responses towards induced plants. Overall, herbivores showed little preference for undamaged or induced plants, even though deterrence of future herbivory may pose an effective defence strategy to prevent feeding damage (Karban & Baldwin, 1997). The outcomes contrast with the hypothesis that specialist herbivores use volatiles from their induced host plant as cues for oviposition or feeding and are often better able to cope with host plant specific defences, whereas generalist herbivores avoid induced plants and are strongly affected by host plant‐specific defences (Ali & Agrawal, 2012). The surprising lack of a significant preference for undamaged plants by generalists in our meta‐analysis may be attributed to the method of measuring preference. Although oviposition preferences were generally higher for undamaged plants, feeding preferences were similar for undamaged and induced plants. The studies included in this meta‐analysis are biased towards insect herbivores that rely on oviposition by the female for their host plant choice (Jaenike, 1978). This suggests that no strong individual feeding choice may have evolved in such herbivores, because larval insect herbivores rarely get a chance to choose their host plant and might therefore not be able to pick the most suitable host plant. Alternatively, preference under artificial conditions may not reflect the choices that herbivores make in natural settings. Our results confirm that the magnitude of induced resistance was stronger in glasshouse studies than in field experiments, suggesting that other factors such as resource availability and simultaneous other stressors modulate plant responses and may result in altered herbivore preference (Joern *et al*., 2012). The efficacy of herbivore deterrence as an induced resistance strategy may thus depend on the study system and experimental set‐up, which should be considered carefully in predicting plant defence strategies (Gong & Zhang, 2014).

Although the host plant identity may be important in predicting plant responses to herbivory (Garcia *et al*., 2021), we found that mean plant performance, herbivore performance and preference vary little across plant life histories. Overall, a large portion of variation in plant performance remained unexplained, suggesting that plant defence strategies can differ greatly depending on the species. This requires placing the component of induced resistance in the context of the entire strategy that plants use to deal with herbivory, as well as abiotic stress, competition and beneficial interactions such as pollination. Plants may utilize defence strategies besides induced direct defence, including constitutively expressed resistance traits, (over)compensation, tolerance or even escape from herbivory by life history or apparency (Kant *et al*., 2015; Garcia & Eubanks, 2019; Karban, 2020). Moreover, plants may be constrained by trade‐offs between various defence mechanisms due to the associated costs. Trade‐offs between constitutive and induced defence have been found within and across plant species and may represent alternative defence strategies depending on the species' environment (Kempel *et al*., 2011; Agrawal & Hastings, 2019). Furthermore, the optimal defence hypothesis predicts that plants allocate their defence budget to the most valuable tissue (Strauss *et al*., 2002; Karban, 2020). Predicting the outcome of a multiherbivore attack may thus strongly depend on whether multiple herbivores feed on the same tissue, whether the tissues are closely connected by the sap stream and when the attacks occur over the plants' ontogeny (Barton & Koricheva, 2010; Moreira *et al*., 2018). Lastly, plants' defence strategies to sequential herbivore attacks may have evolved with specific herbivore communities, rather than herbivore species in isolation, which means that our ecological predictors for plant defence strategies may be found in specific combinations between plant species and their environment (Mertens *et al*., 2021). Most studies on plant interactions with multiple herbivores do not report characteristics of the broader defence strategies mentioned here for the plant species studied. We could therefore not test the hypothesis that responses to sequential attack are predicted by the overall defence strategy.

This meta‐analysis reveals a major knowledge gap when it comes to the impact of sequential herbivore attack on plant fitness. Plant performance is usually reported as biomass gain or tissue loss and can indicate resistance to herbivores, but it does not show the adaptive value of induced defence. Besides, many plant–herbivore studies are performed on crops, in which the main goal is often not to maximize plant fitness, but the yield of the entire stand, which is usually defined as high biomass with low damage levels for leafy crops. Therefore, our results regarding induced resistance might be useful in agriculture, but they teach us little about the plant fitness of wild plant species (Younginger *et al*., 2017; Poelman *et al*., 2023). To understand the evolution of induced defences and their adaptive value compared with other defence strategies, experiments should measure the reproductive capacity of plants. Also, we should consider plant defence as a set of defence mechanisms and may need parameters for each type of defence to successfully predict plant responses to sequential herbivore attack. Ultimately, our findings show that the plant–herbivore research field should look beyond plant physiology to predict plant defence to multiherbivore attack. Integrating hypotheses on the effects of herbivore identity, environmental conditions and plant ontogeny may strengthen our predictions of plant defence strategies. Studying the integrated response of plants and the resulting reproductive output should be considered to fully understand how plants have evolved to successfully persist in multi‐herbivore environments.

## Competing interests

None declared.

## Author contributions

ZD, JK and EHP designed the research. ZD performed the research and analysed the data. JK and EHP advised about data analysis. ZD, JK and EHP wrote the paper.

## Disclaimer

The New Phytologist Foundation remains neutral with regard to jurisdictional claims in maps and in any institutional affiliations.

## Supporting information


**Fig. S1** PRISMA diagram of the meta‐analysis.
**Fig. S2** Number of effect sizes of plant and herbivore species included in meta‐analysis.
**Fig. S3** Cumulative meta‐analysis.
**Fig. S4** Funnel plots.
**Fig. S5** Validation with additional data.
**Notes S1** References of studies from which data was extracted for the meta‐analysis.

**Table S1** Mean effects when accounting for phylogeny.
**Table S2** Mean effects when excluding most represented species.
**Table S3** Data extracted from the literature for the meta‐analysis.
Please note: Wiley is not responsible for the content or functionality of any Supporting Information supplied by the authors. Any queries (other than missing material) should be directed to the *New Phytologist* Central Office.

## Data Availability

The data that supports the findings of this study is available in Table S3.
