# Supplementary material for: Ecological predictors of plant responses to sequential herbivory: a meta‐analysis
Source: New Phytol. 2025 Dec 17;250(2):1128–40. doi: 10.1111/nph.70822 (PMC13000986; doi:10.1111/nph.70822)
Supplement: Supplementary file 1 — Fig. S1 PRISMA diagram of the meta‐analysis. Fig. S2 Number of effect sizes of plant and herbivore species included in meta‐analysis. Fig. S3 Cumulative meta‐analysis. Fig. S4 Funnel plots. Fig. S5 Validation with additional data. Notes S1 References of studies from which data was extracted for the meta‐analysis. [file NPH-250-1128-s002.pdf]

New Phytologist

***New Phytologist* Supporting Information**

Article title: Ecological predictors of plant responses to sequential herbivory: a meta-analysis
Authors: Zoë Delamore, Julia Koricheva, Erik H. Poelman
Article acceptance date: 30 October 2025

The following Supporting Information is available for this article:

Figure S1 PRISMA diagram of the meta-analysis.

Figure S2 Number of effect sizes of plant and herbivore species included in meta-analysis.

Figure S3 Cumulative meta-analysis.

Figure S4 Funnel plots.

Figure S5 Validation with additional data.

Table S1 Mean effects when accounting for phylogeny.

Table S2 Mean effects when excluding most represented species.

Table S3 Data extracted from the literature for the meta-analysis (submitted separately).

Notes S1 References of studies from which data was extracted for the meta-analysis.

Fig. S1 PRISMA diagram of the meta-analysis

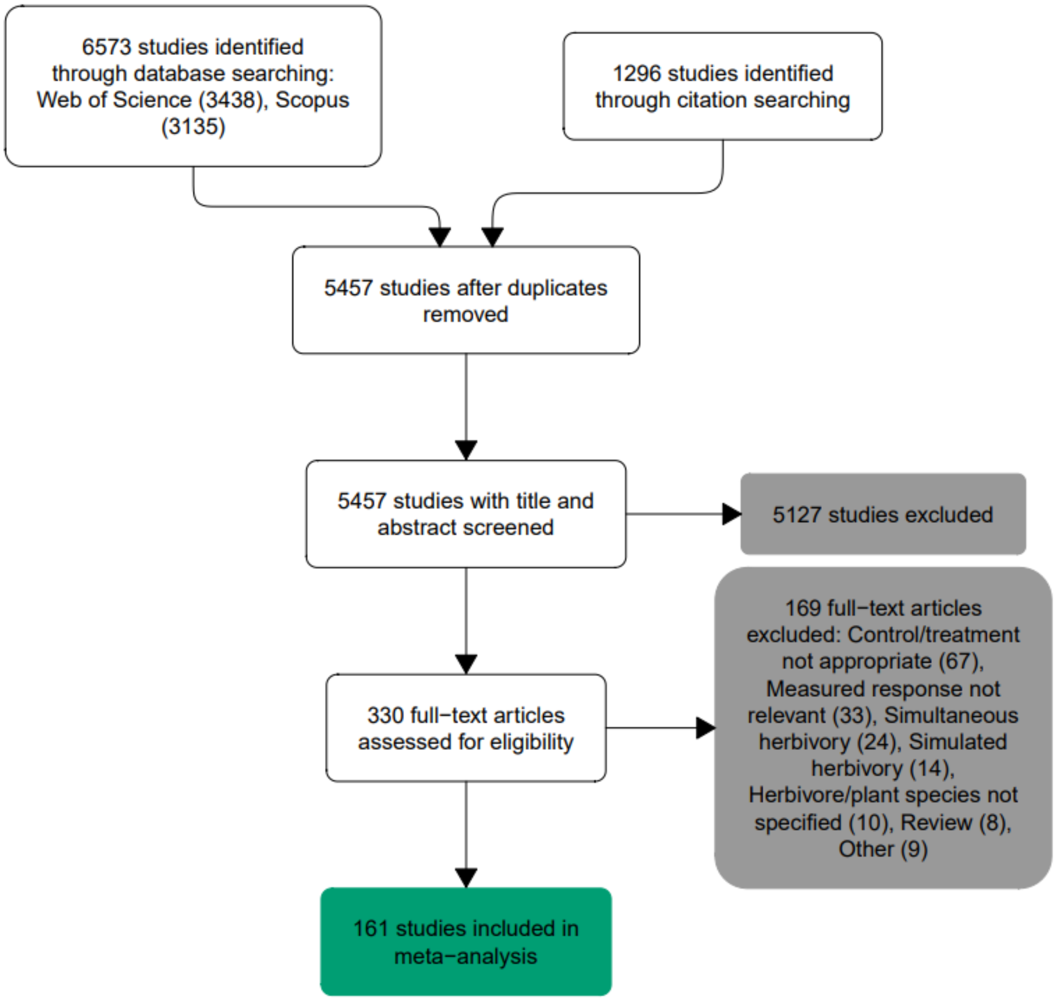

Fig. S2 Number of effect sizes of each plant (A), inducing herbivore (B) and subsequent herbivore (C) species in the meta-analysis.

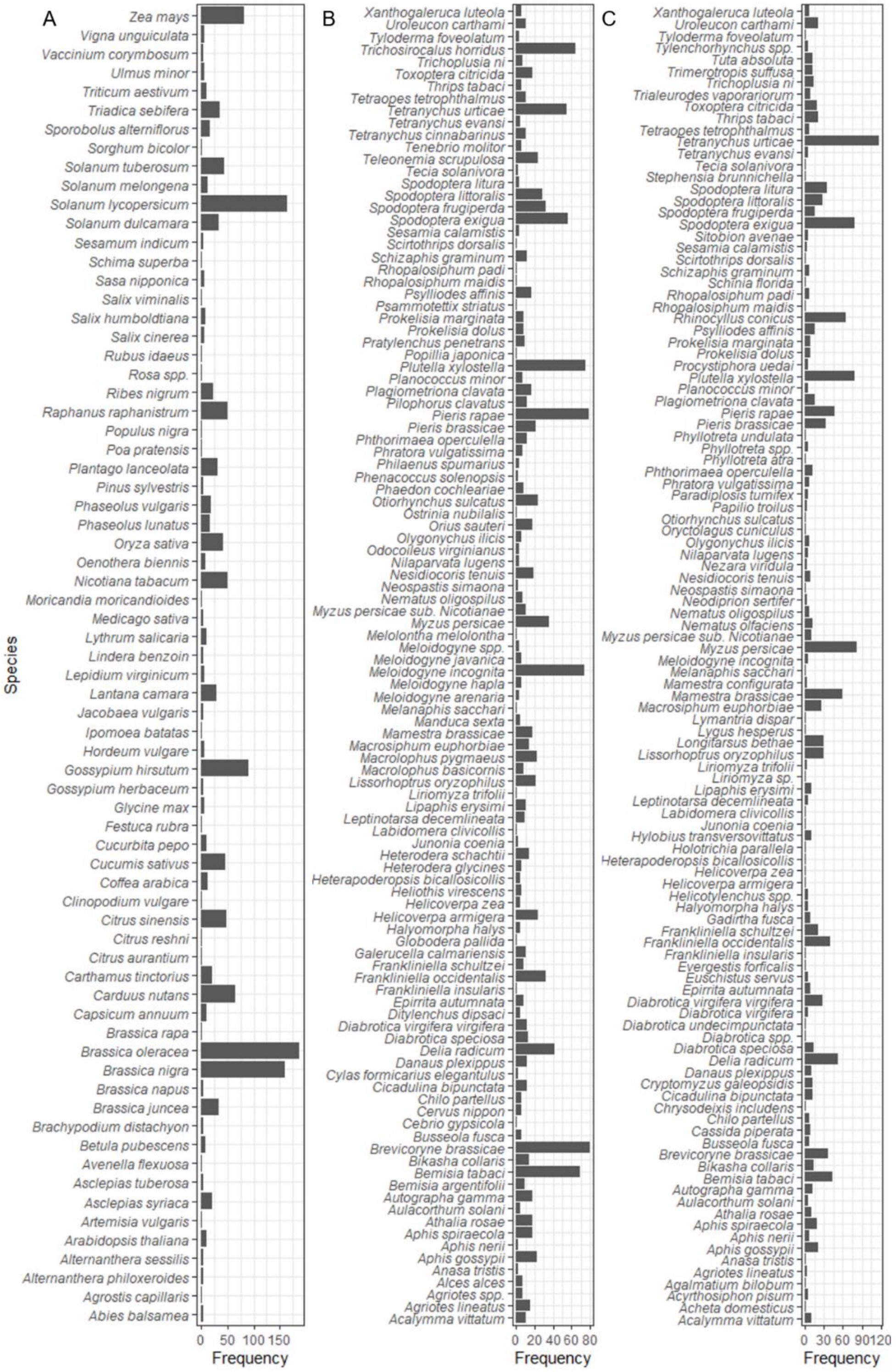

Fig. S3 Cumulative meta-analysis by publication year of effects of sequential herbivory on herbivore performance (A), herbivore preference (B) and plant performance (C).

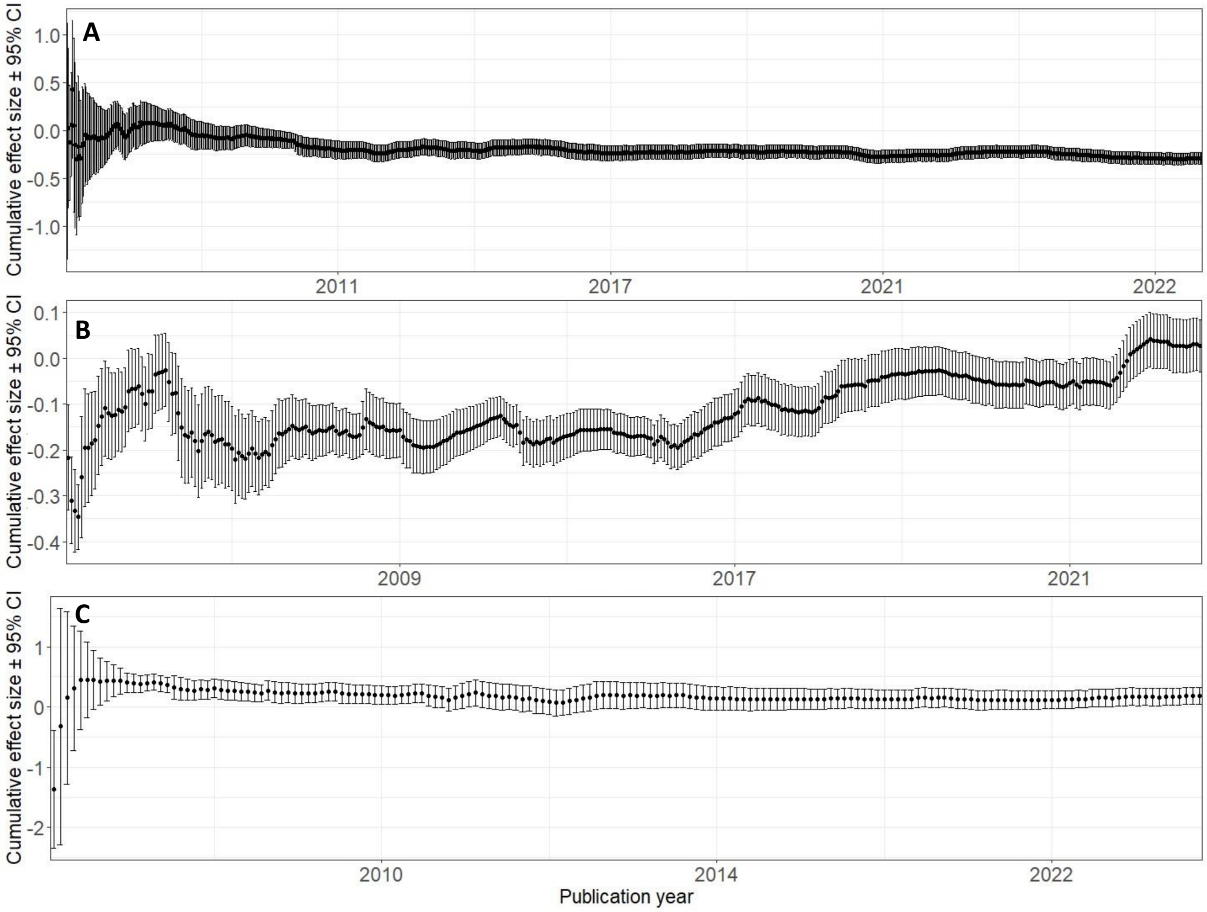

Fig. S4 Figure S4. Funnel plots for effects of sequential herbivory on herbivore performance (A), herbivore preference (B) and plant performance (C).

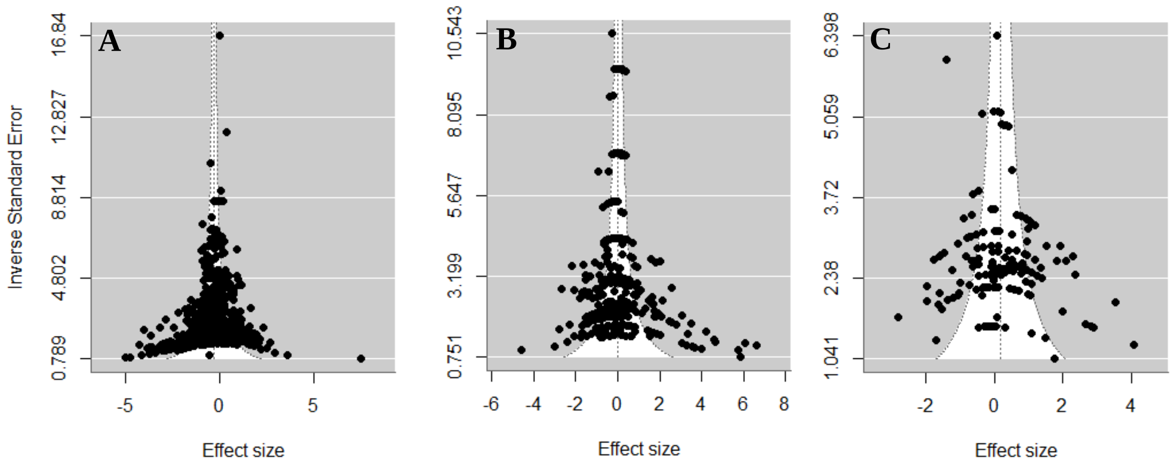

Fig. S5 Orchard plot showing the mean effect size (log odds ratio) for effects of sequential herbivory on herbivore performance (A) and preference (B). Bold lines show 95% confidence interval (CI); thin lines the 95% prediction interval (PI); coloured points the individual effect sizes. k = the number of effects included, with the number of studies between brackets.

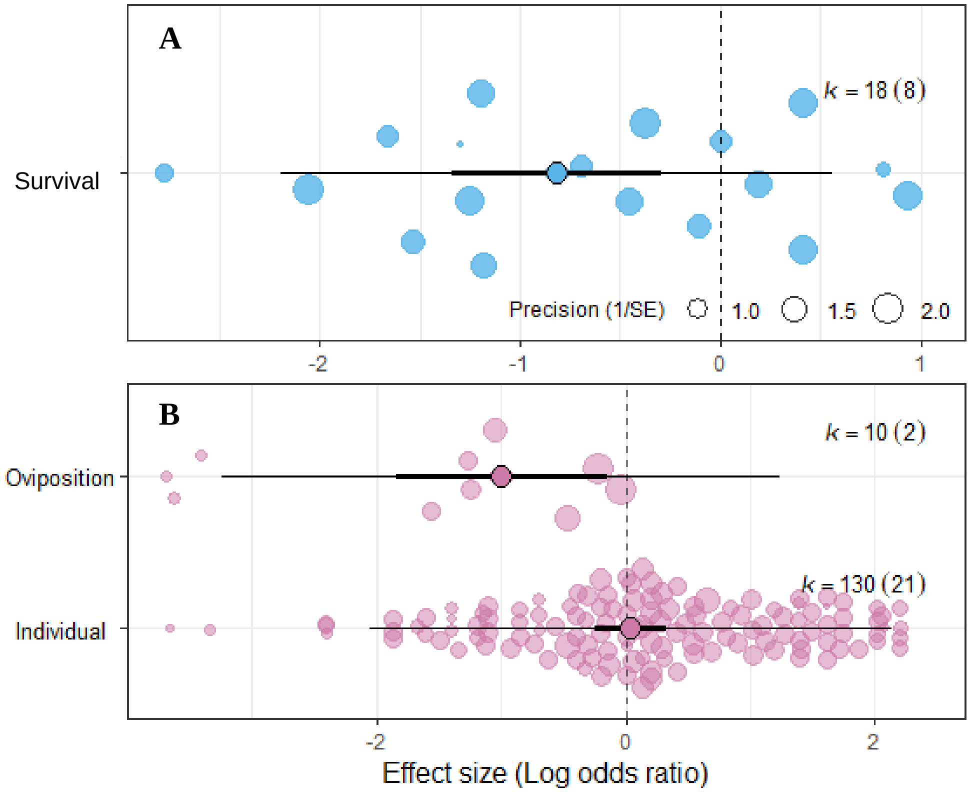

Table S1 Test results of single moderator models with and without plant or herbivore phylogeny as a random factor. Bold values indicate differences in significance of moderators as compared to the main analysis (without accounting for phylogeny).

		Without accounting for phylogeny				Accounting for plant phylogeny				Accounting for inducing herbivore phylogeny				Accounting for subsequent herbivore phylogeny			
		k	Qm	df	p	K	Qm	df	p	k	Qm	df	p	k	Qm	df	p
Herbivore performance	Plant life history	834	1.183	3	0.757	834	1.183	3	0.757	659	2.404	3	0.493	707	1.803	3	0.614
	Plant cultivation	834	0.855	1	0.355	834	0.855	1	0.355	659	1.143	1	0.285	707	0.508	1	0.476
	Feeding guild	834	29.71	13	0.005	834	29.71	13	0.005	**659**	**15.89**	**10**	**0.103**	707	31.27	12	0.002
	Diet breadth	834	1.029	3	0.794	834	1.029	3	0.794	659	3.914	3	0.271	707	1.515	3	0.679
	Feeding location	834	0.768	3	0.857	834	0.768	3	0.857	659	1.406	3	0.704	707	1.203	3	0.752
	1st spp = 2nd spp	834	1.212	1	0.271	834	1.212	1	0.271	659	0.653	1	0.419	707	1.715	1	0.190
	1st spp present	816	1.875	1	0.171	816	1.875	1	0.171	652	0.435	1	0.51	698	2.401	1	0.121
	Experiment type	834	7.486	1	0.006	834	7.486	1	0.006	659	7.255	1	0.007	707	6.667	1	0.010
	Duration 1st attack	804	0.313	1	0.576	**831**	**4.001**	**1**	**0.045**	656	3.051	1	0.081	704	2.208	1	0.137
	Duration 2nd attack	684	1.986	1	0.159	**831**	**4.425**	**1**	**0.035**	**656**	**4.982**	**1**	**0.026**	704	2.052	1	0.152
Herbivore preference	Plant life history	339	3.220	3	0.359	339	3.220	3	0.359	292	2.079	3	0.556	294	2.327	3	0.507
	Plant cultivation	339	0.694	1	0.405	339	0.694	1	0.405	292	0.529	1	0.467	294	0.207	1	0.649
	Feeding guild	339	9.645	13	0.723	339	9.645	13	0.723	292	6.381	9	0.701	294	8.445	11	0.673
	Diet breadth	339	9.874	3	0.02	339	9.874	3	0.02	**292**	**3.565**	**3**	**0.312**	294	9.53	3	0.023
	Feeding location	339	19.25	3	<0.001	339	19.25	3	<0.001	292	14.51	3	0.002	294	22.26	3	<0.001
	1st spp = 2nd spp	339	1.112	1	0.292	339	1.112	1	0.292	292	0.127	1	0.722	294	1.687	1	0.194
	1st spp present	331	0.496	1	0.481	331	0.496	1	0.481	288	0.690	1	0.406	289	0.309	1	0.579
	Experiment type	339	0.782	1	0.376	339	0.782	1	0.376	292	0.002	1	0.963	294	0.049	1	0.825
	Duration 1st attack	331	1.866	1	0.172	336	1.623	1	0.203	289	0.290	1	0.590	291	0.005	1	0.941
	Duration 2nd attack	252	0.048	1	0.826	339	0.441	1	0.506	292	0.324	1	0.569	294	0.399	1	0.527
Plant performance	Plant life history	172	0.716	3	0.869	172	0.182	3	0.980	129	0.613	3	0.893	135	3.412	3	0.332
	Plant cultivation	172	0.006	1	0.938	172	0.001	1	0.989	129	0.424	1	0.515	135	2.819	1	0.093
	Feeding guild	172	36.73	11	<0.001	172	36.12	11	<0.001	129	35.09	8	<0.001	135	32.79	8	<0.001
	Diet breadth	172	22.61	3	<0.001	172	22.61	3	<0.001	129	22.59	3	<0.001	135	20.32	3	<0.001
	Feeding location	172	6.513	3	0.111	172	5.851	3	0.119	**129**	**9.311**	**2**	**0.01**	135	2.935	3	0.402
	1st spp = 2nd spp	172	0.496	1	0.43	172	0.798	1	0.372	129	0.449	1	0.503	135	0.490	1	0.484
	1st spp present	167	2.237	1	0.029	**167**	**1.992**	**1**	**0.166**	128	4.367	1	0.037	**131**	**0.735**	**1**	**0.391**
	Experiment type	172	0.258	1	0.926	172	0.258	1	0.926	129	0.464	1	0.496	135	0.093	1	0.761
	Duration 1st attack	162	1.866	1	0.908	162	1.805	1	0.179	129	2.687	1	0.101	135	0.059	1	0.808
	Duration 2nd attack	165	0.992	1	0.319	165	0.661	1	0.416	129	2.331	1	0.127	135	0.255	1	0.614

Table S2 Mean effects (±95% CI) where one of the most represented species was excluded each time for each of the responses measured. Values are printed in bold when confidence intervals differ from the main analysis (no species excluded). For response variables indicated with an asterisk the sign of the effect has been reversed to ensure consistent interpretation of measured responses (i.e. positive effects indicate higher herbivore or plant performance following sequential herbivory)

Species excluded		Herbivore performance			
		Survival	Mass/size	Fecundity	Development time*
	None	-0.14 [-0.36, 0.08]	-0.33 [-0.49, -0.18]	-0.35 [-0.53, -0.17]	-0.46 [-0.69, -0.23]
Plant species	*Brassica oleracea*	-0.12 [-0.37, 0.13]	-0.37 [-0.55, -0.2]	-0.36 [-0.55, -0.16]	-0.58 [-0.88, -0.28]
	Brassica nigra	-0.15 [-0.37, 0.08]	-0.34 [-0.5, -0.17]	-0.35 [-0.54, -0.16]	-0.48 [-0.72, -0.23]
	Solanum lycopersicum	-0.15 [-0.39, 0.1]	-0.33 [-0.49, -0.16]	-0.3 [-0.5, -0.09]	-0.44 [-0.69, -0.18]
Inducing herbivore	*Brevicoryne brassicae*	-0.14 [-0.36, 0.08]	-0.36 [-0.52, -0.2]	-0.35 [-0.53, -0.18]	-0.5 [-0.75, -0.26]
	Plutella xylostella	-0.14 [-0.36, 0.08]	-0.32 [-0.48, -0.16]	-0.36 [-0.54, -0.18]	-0.46 [-0.7, -0.23]
	Meloidogyne incognita	-0.13 [-0.36, 0.1]	-0.35 [-0.51, -0.18]	-0.35 [-0.54, -0.16]	-0.46 [-0.71, -0.21]
Subsequent herbivore	*Tetranychus urticae*	-0.13 [-0.36, 0.1]	-0.32 [-0.48, -0.17]	-0.3 [-0.49, -0.11]	-0.45 [-0.68, -0.22]
	Myzus persicae	-0.03 [-0.25, 0.19]	-0.31 [-0.46, -0.16]	-0.27 [-0.45, -0.09]	-0.42 [-0.65, -0.2]
	Plutella xylostella	-0.14 [-0.37, 0.09]	-0.35 [-0.52, -0.19]	-0.34 [-0.53, -0.16]	-0.52 [-0.78, -0.26]

Species excluded		Herbivore preference		Plant performance		
		Oviposition	Individual	Nr of seeds/fruits	Damage*	Biomass
	None	-0.33 [-0.68, 0.02]	-0.05 [-0.33, 0.24]	0.09 [-0.73, 0.9]	0.25 [0.02, 0.48]	-0.33 [-0.6, -0.06]
Plant species	*Brassica oleracea*	**-0.34 [-0.63, -0.04]**	-0.12 [-0.33, 0.09]	0.07 [-0.75, 0.89]	0.24 [0.01, 0.48]	-0.36 [-0.65, -0.08]
	Brassica nigra	-0.38 [-0.77, 0.01]	0.01 [-0.31, 0.32]	0.08 [-0.74, 0.9]	0.25 [0.02, 0.48]	-0.35 [-0.62, -0.07]
	Solanum lycopersicum	-0.35 [-0.71, 0.01]	-0.04 [-0.33, 0.25]	0.07 [-0.75, 0.9]	0.25 [0.01, 0.49]	-0.36 [-0.64, -0.07]
Inducing herbivore	*Brevicoryne brassicae*	-0.36 [-0.73, 0]	-0.04 [-0.34, 0.25]	0.09 [-0.73, 0.9]	0.25 [0.02, 0.48]	-0.33 [-0.6, -0.06]
	Plutella xylostella	-0.34 [-0.68, 0.01]	-0.06 [-0.33, 0.21]	-0.08 [-0.79, 0.64]	**0.1 [-0.15, 0.35]**	-0.34 [-0.62, -0.07]
	Meloidogyne incognita	-0.33 [-0.68, 0.03]	-0.03 [-0.32, 0.25]	0.12 [-0.69, 0.92]	0.27 [0.05, 0.49]	-0.43 [-0.69, -0.16]
Subsequent herbivore	*Tetranychus urticae*	-0.35 [-0.71, 0.01]	-0.12 [-0.42, 0.17]	0.08 [-0.74, 0.89]	0.24 [0, 0.48]	-0.33 [-0.6, -0.06]
	Myzus persicae	-0.32 [-0.68, 0.04]	-0.03 [-0.33, 0.27]	0.11 [-0.69, 0.92]	0.26 [0.04, 0.49]	**-0.26 [-0.54, 0.01]**
	Plutella xylostella	-0.34 [-0.7, 0.01]	-0.03 [-0.31, 0.25]	0.02 [-0.73, 0.76]	0.25 [0, 0.5]	-0.32 [-0.61, -0.03]

Table S3 Data extracted from the literature for the meta-analysis (submitted separately). Large table containing all data included in the meta-analysis, with each row representing the data extracted from one experiment. In some scientific publications, multiple relevant experiments were included and are therefore represented by multiple rows in the dataset.

Notes S1 References of studies from which data was extracted for the meta-analysis.

Agrawal AA. **1998**. Induced Responses to Herbivory and Increased Plant Performance. *Science* **279**: 1201–1202.

Agrawal AA. **1999**. Induced responses to herbivory in wild radish: Effects on several herbivores and plant fitness. *Ecology* **80**: 1713–1723.

Agrawal A. **2000**. Benefits and Costs of Induced Plant Defense for Lepidium virginicum (Brassicaceae).

Agrawal AA, Gorski PM, Tallamy DW. **1999**. Polymorphism in plant defence agianst herbivory: constitutive and induced resistance in Cucumis sativus.

Agrawal AA, Sherriffs MF. **2001**. Induced Plant Resistance and Susceptibility to Late-Season Herbivores of Wild Radish. *Annals of the Entomological Society of America* **94**: 71–75.

Agut B, Gamir J, Jaques JA, Flors V. **2015**. *T etranychus urticae* -triggered responses promote genotype-dependent conspecific repellence or attractiveness in citrus. *New Phytologist* **207**: 790–804.

Ali JG, Agrawal AA. **2014**. Asymmetry of plant-mediated interactions between specialist aphids and caterpillars on two milkweeds (C Fox, Ed.). *Functional Ecology* **28**: 1404–1412.

Amiri Domari M, Mansouri SM, Mehrparvar M. **2021**. Previous herbivory modulates aphid population growth and plant defense responses in a non-model plant, *Carthamus tinctorius* (Asteraceae). *Bulletin of Entomological Research* **111**: 715–725.

Anderson P, Sadek MM, Wäckers FL. **2011**. Root herbivory affects oviposition and feeding behavior of a foliar herbivore. *Behavioral Ecology* **22**: 1272–1277.

Arce CCM, Machado RAR, Ribas NS, Cristaldo PF, Ataíde LMS, Pallini Â, Carmo FM, Freitas LG, Lima E. **2017**. Nematode Root Herbivory in Tomato Increases Leaf Defenses and Reduces Leaf Miner Oviposition and Performance. *Journal of Chemical Ecology* **43**: 120–128.

Bezemer TM, Wagenaar R, Van Dam NM, Wäckers FL. **2003**. Interactions between above- and belowground insect herbivores as mediated by the plant defense system. *Oikos* **101**: 555–562.

Biru FN, Cazzonelli CI, Elbaum R, Johnson SN. **2022**. Contrasting impacts of herbivore induction and elevated atmospheric CO_2 on silicon defences and consequences for subsequent herbivores. *Entomologia Experimentalis et Applicata* **170**: 681–688.

Bustos-Segura C, Cuny MAC, Benrey B. **2020**. Parasitoids of leaf herbivores enhance plant fitness and do not alter caterpillar-induced resistance against seed beetles (K Mooney, Ed.). *Functional Ecology* **34**: 586–596.

Chen J, Chen X, Stout MJ, Davis JA. **2022**. Belowground Herbivory to Sweetpotato by Sweetpotato Weevil (Coleoptera: Brentidae) Alters Population Dynamics and Probing Behavior of Aboveground Herbivores (A Rashed, Ed.). *Journal of Economic Entomology* **115**: 1069–1075.

Clark KE, Hartley SE, Johnson SN. **2011**. Does mother know best? The preference-performance hypothesis and parent-offspring conflict in aboveground-belowground herbivore life cycles. *Ecological Entomology* **36**: 117–124.

Coppola V, Soler R, Rao R, Corrado G. **2017**. Tomato-mediated interactions between root herbivores and aphids: insights into plant defence signalling. *Entomologia Experimentalis et Applicata* **163**: 170–176.

Costa EN, Fernandes MG, Reis LC, Martins LO, Foresti AC, De Paula Quintão Scalon S. **2022**. Above- and belowground resistance in Brazilian maize varieties under attack of *Spodoptera frugiperda* and *Diabrotica speciosa*. *Entomologia Experimentalis et Applicata* **170**: 718–726.

Dahmane M, Urbaneja A, Ruíz-Rivero O, Alonso-Valiente M, Pérez-Hedo M. **2022**. The zoophytophagous predator Pilophorus clavatus (Hemiptera: Miridae) induces plant defences in citrus. *Journal of Pest Science*.

Dalin P, Björkman C. **2003**. Adult beetle grazing induces willow trichome defence against subsequent larval feeding. *Oecologia* **134**: 112–118.

Davidson-Lowe E, Ali JG. **2021**. Herbivore-induced plant volatiles mediate behavioral interactions between a leaf-chewing and a phloem-feeding herbivore. *Basic and Applied Ecology* **53**: 39–48.

Davidson-Lowe E, Szendrei Z, Ali JG. **2019**. Asymmetric effects of a leaf-chewing herbivore on aphid population growth. *Ecological Entomology* **44**: 81–92.

De Oliveira EF, Pallini A, Janssen A. **2019**. Herbivore performance and plant defense after sequential attacks by inducing and suppressing herbivores. *Insect Science* **26**: 108–118.

Delphia CM, Mescher MC, De Moraes CM. **2007**. Induction of Plant Volatiles by Herbivores with Different Feeding Habits and the Effects of Induced Defenses on Host-Plant Selection by Thrips. *Journal of Chemical Ecology* **33**: 997–1012.

Denno RF, Peterson MA, Gratton C, Cheng J, Langellotto GA, Huberty AF, Finke DL. **2000**. Feeding-induced changes in plant quality mediate interspecific competition between sap-feeding herbivores. *Ecology* **81**: 1814–1827.

Di N, Zhu Z, Harwood JD, Xu Z, Wang S, Desneux N. **2022**. Fitness of Frankliniella occidentalis and Bemisia tabaci on three plant species pre-inoculated by Orius sauteri. *Journal of Pest Science* **95**: 1531–1541.

Eisenring M, Glauser G, Meissle M, Romeis J. **2018**. Differential Impact of Herbivores from Three Feeding Guilds on Systemic Secondary Metabolite Induction, Phytohormone Levels and Plant-Mediated Herbivore Interactions. *Journal of Chemical Ecology* **44**: 1178–1189.

Eisenring M, Naranjo SE, Bacher S, Abbott A, Meissle M, Romeis J. **2019**. Reduced caterpillar damage can benefit plant bugs in Bt cotton. *Scientific Reports* **9**: 2727.

Erb M, Flors V, Karlen D, De Lange E, Planchamp C, D'Alessandro M, Turlings TCJ, Ton J. **2009**. Signal signature of aboveground-induced resistance upon belowground herbivory in maize. *The Plant Journal* **59**: 292–302.

Erb M, Robert CAM, Hibbard BE, Turlings TCJ. **2011a**. Sequence of arrival determines plant-mediated interactions between herbivores. *Journal of Ecology* **99**: 7–15.

Erb M, Robert CAM, Marti G, Lu J, Doyen G, Villard N, Barrière Y, French BW, Wolfender J-L, Turlings T, *et al.* **2015**. A physiological and behavioral mechanism for leaf-herbivore induced systemic root resistance. *Plant Physiology*: pp.00759.2015.

Erb M, Robert CAM, Turlings TCJ. **2011b**. Induction of root-resistance by leaf-herbivory follows a vertical gradient. *Journal of Plant Interactions* **6**: 133–136.

Erwin AC, Züst T, Ali JG, Agrawal AA. **2014**. Above-ground herbivory by red milkweed beetles facilitates above- and below-ground conspecific insects and reduces fruit production in common milkweed (K Whitney, Ed.). *Journal of Ecology* **102**: 1038–1047.

Escobar-Bravo R, Klinkhamer PGL, Leiss KA. **2017**. Induction of Jasmonic Acid-Associated Defenses by Thrips Alters Host Suitability for Conspecifics and Correlates with Increased Trichome Densities in Tomato. *Plant and Cell Physiology* **58**: 622–634.

Escobar-Bravo R, Schimmel BCJ, Glauser G, Klinkhamer PGL, Erb M. **2022**. Leafminer attack accelerates the development of soil-dwelling conspecific pupae via plant-mediated changes in belowground volatiles. *New Phytologist* **234**: 280–294.

Esmaeily S, Amin Samih M, Izadi H. **2020**. Induced eggplant resistance against *Trialeurodes vaporariorum* triggered by jasmonic acid, abscisic acid, and *Nesidiocoris tenuis* feeding. *Bulletin of Entomological Research* **110**: 285–292.

Franzin ML, Coffler Botti JM, Matiello Fadini MA, Ferreira Melo JO, Mendes SM. **2020**. Multiple Infestations Induce Direct Defense of Maize to Tetranychus urticae (Acari: Tetranychidae). *Florida Entomologist* **103**.

Gange AC, Brown VK. **1989**. Effects of root herbivory by an insect on a foliar-feeding species, mediated through changes in the host plant. *Oecologia* **81**: 38–42.

Gao J, Arthurs S, Mao R. **2020**. Asymmetric Interaction between Aphis spiraecola and Toxoptera citricida on Sweet Orange Induced by Pre-Infestation. *Insects* **11**: 414.

Guo H, Ge F. **2017**. Root nematode infection enhances leaf defense against whitefly in tomato. *Arthropod-Plant Interactions* **11**: 23–33.

Hol WHG. **2016**. Root-Lesion Nematodes Suppress Cabbage Aphid Population Development by Reducing Aphid Daily Reproduction. *Frontiers in Plant Science* **7**.

Hol WHG, De Boer W, Termorshuizen AJ, Meyer KM, Schneider JHM, Van Der Putten WH, Van Dam NM. **2013**. Heterodera schachtii Nematodes Interfere with Aphid-Plant Relations on Brassica oleracea. *Journal of Chemical Ecology* **39**: 1193–1203.

Hoysted GA, Lilley CJ, Field KJ, Dickinson M, Hartley SE, Urwin PE. **2017**. A Plant-Feeding Nematode Indirectly Increases the Fitness of an Aphid. *Frontiers in Plant Science* **8**: 1897.

Huang W, Robert CAM, Hervé MR, Hu L, Bont Z, Erb M. **2017**. A mechanism for sequence specificity in plant-mediated interactions between herbivores. *New Phytologist* **214**: 169–179.

Huang W, Siemann E, Xiao L, Yang X, Ding J. **2014**. Species-specific defence responses facilitate conspecifics and inhibit heterospecifics in above–belowground herbivore interactions. *Nature Communications* **5**: 4851.

Hunt-Joshi TR, Blossey B. **2005**. Interactions of root and leaf herbivores on purple loosestrife (Lythrum salicaria). *Oecologia* **142**: 554–563.

Jiang J, Xu L-L, Yu W-Y, Zhang S-Z, Liu T-X. **2022**. Improvement of Bemisia tabaci (Hemiptera: Aleyrodidae) Fitness on Chinese Kale upon Simultaneous Herbivory by Plutella xylostella (Lepidoptera: Plutellidae). *Biology* **11**: 72.

Johnson SN, Hawes C, Karley AJ. **2009**. Reappraising the role of plant nutrients as mediators of interactions between root- and foliar-feeding insects. *Functional Ecology* **23**: 699–706.

Johnson SN, Mitchell C, McNicol JW, Thompson J, Karley AJ. **2013**. Downstairs drivers - root herbivores shape communities of above-ground herbivores and natural enemies via changes in plant nutrients (J Newman, Ed.). *Journal of Animal Ecology* **82**: 1021–1030.

Kafle D, Hänel A, Lortzing T, Steppuhn A, Wurst S. **2017**. Sequential above- and belowground herbivory modifies plant responses depending on herbivore identity. *BMC Ecology* **17**: 5.

Kammerhofer N, Egger B, Dobrev P, Vankova R, Hofmann J, Schausberger P, Wieczorek K. **2015**. Systemic above- and belowground cross talk: hormone-based responses triggered by *Heterodera schachtii* and shoot herbivores in *Arabidopsis thaliana*. *Journal of Experimental Botany* **66**: 7005–7017.

Kaplan I, Sardanelli S, Denno RF. **2009**. Field evidence for indirect interactions between foliar-feeding insect and root-feeding nematode communities on *Nicotiana tabacum*. *Ecological Entomology* **34**: 262–270.

Kaplan I, Sardanelli S, Rehill BJ, Denno RF. **2011**. Toward a mechanistic understanding of competition in vascular-feeding herbivores: an empirical test of the sink competition hypothesis. *Oecologia* **166**: 627–636.

Karssemeijer PN, Reichelt M, Gershenzon J, Van Loon J, Dicke M. **2020**. Foliar herbivory by caterpillars and aphids differentially affects phytohormonal signalling in roots and plant defence to a root herbivore. *Plant, Cell & Environment* **43**: 775–786.

Karssemeijer PN, Winzen L, Van Loon JJA, Dicke M. **2022**. Leaf-chewing herbivores affect preference and performance of a specialist root herbivore. *Oecologia* **199**: 243–255.

Kempel A, Schmidt AK, Brandl R, Schädler M. **2010**. Support from the underground: Induced plant resistance depends on arbuscular mycorrhizal fungi: Mycorrhiza and induced resistance. *Functional Ecology* **24**: 293–300.

Kiełkiewicz M, Barczak-Brzyżek A, Karpińska B, Filipecki M. **2019**. Unravelling the Complexity of Plant Defense Induced by a Simultaneous and Sequential Mite and Aphid Infestation. *International Journal of Molecular Sciences* **20**: 806.

Kostenko O, Mulder PPJ, Bezemer TM. **2013**. Effects of Root Herbivory on Pyrrolizidine Alkaloid Content and Aboveground Plant-Herbivore-Parasitoid Interactions in Jacobaea Vulgaris. *Journal of Chemical Ecology* **39**: 109–119.

Kraus EC, Stout MJ. **2019a**. Effects of defoliation on the resistance and tolerance of rice, *Oryza sativa,* to root injury by the rice water weevil, *Lissorhoptrus oryzophilus*. *Entomologia Experimentalis et Applicata* **167**: 350–359.

Kraus EC, Stout MJ. **2019b**. Plant-mediated interactions among above-ground and below-ground life stages of a root-feeding weevil. *Ecological Entomology* **44**: 771–779.

Kroes A, Stam JM, David A, Boland W, van Loon JJA, Dicke M, Poelman EH. **2016**. Plant-mediated interactions between two herbivores differentially affect a subsequently arriving third herbivore in populations of wild cabbage. *Plant Biology* **18**: 981–991.

Kumar P, Ortiz EV, Garrido E, Poveda K, Jander G. **2016**. Potato tuber herbivory increases resistance to aboveground lepidopteran herbivores. *Oecologia* **182**: 177–187.

Kutyniok M, Persicke M, Müller C. **2014**. Effects of Root Herbivory by Nematodes on the Performance and Preference of a Leaf-Infesting Generalist Aphid Depend on Nitrate Fertilization. *Journal of Chemical Ecology* **40**: 118–127.

Lackner S, Lackus ND, Paetz C, Köllner TG, Unsicker SB. **2019**. Aboveground phytochemical responses to belowground herbivory in poplar trees and the consequence for leaf herbivore preference. *Plant, Cell & Environment* **42**: 3293–3307.

Li Y, Dicke M, Harvey JA, Gols R. **2014**. Intra-specific variation in wild Brassica oleracea for aphid-induced plant responses and consequences for caterpillar–parasitoid interactions. *Oecologia* **174**: 853–862.

Li X, Li B, Meng L. **2017**. Below-ground nematode herbivory of resistant soybean cultivars impairs the performances of an above-ground caterpillar and its parasitoid. *Ecological Entomology* **42**: 712–720.

Li Y, Meijer D, Dicke M, Gols R. **2018**. Oviposition preference of three lepidopteran species is not affected by previous aphid infestation in wild cabbage. *Entomologia Experimentalis et Applicata* **166**: 402–411.

Li Y, Qu C, Yan X, Sun X, Yin Z, Zhao H. **2022**. Effect of Feeding Stage and Density of Whiteflies on Subsequent Aphid Performance on Tobacco Plants. *Agronomy* **12**: 1025.

Li Y, Zhen S, Shan S, Sun B, Li J, Hu F, Cui Q, Zhang L, Gu X, Cheng W, *et al.* **2020**. Modulation of above-belowground plant-herbivore interactions by entomopathogenic nematodes. *Applied Soil Ecology* **148**: 103479.

Lin D, Xu Y, Wu H, Liu X, Zhang L, Wang J, Rao Q. **2019**. Plant Defense Responses Induced by Two Herbivores and Consequences for Whitefly Bemisia tabaci. *Frontiers in Physiology* **10**: 346.

Lind EM, Myron EP, Giaccai J, Parker JD. **2012**. White-Tailed Deer Alter Specialist and Generalist Insect Herbivory Through Plant Traits. *Environmental Entomology* **41**: 1409–1416.

Liu Z, Cai Y, Fang Y, Jing J, Li K. **2010**. Induced response in Schima superba: Effects of early- season herbivory on leaf traits and subsequent insect attack.

Liu M, Zhou F, Pan X, Zhang Z, Traw MB, Li B. **2018**. Specificity of herbivore-induced responses in an invasive species, *Alternanthera philoxeroides* (alligator weed). *Ecology and Evolution* **8**: 59–70.

Ma G, Shi X, Kang Z, Gao X. **2018**. The influence of Tetranychus cinnabarinus-induced plant defense responses on Aphis gossypii development. *Journal of Integrative Agriculture* **17**: 164–172.

Magalhães DM, Borges M, Laumann RA, Blassioli Moraes MC. **2018**. Influence of multiple- and single-species infestations on herbivore-induced cotton volatiles and Anthonomus grandis behaviour. *Journal of Pest Science* **91**: 1019–1032.

Mao L, Story RN, Hammond AM, Peterson JK, Labonte DR. **2003**. Effects of Previous Insect Feeding Injury to Sweet Potato on Resistance to Sweet Potato Weevil (Coleoptera: Curculionidae) and Storage Root Chemistry. *Journal of Entomological Science* **38**: 72–83.

Martínez-Medina A, Mbaluto CM, Maedicke A, Weinhold A, Vergara F, Van Dam NM. **2021**. Leaf herbivory counteracts nematode-triggered repression of jasmonate-related defenses in tomato roots. *Plant Physiology* **187**: 1762–1778.

Mathur V, Ganta S, Raaijmakers CE, Reddy AS, Vet LEM, Van Dam NM. **2011**. Temporal dynamics of herbivore-induced responses in Brassica juncea and their effect on generalist and specialist herbivores: Temporal dynamics of induced responses in Brassica juncea. *Entomologia Experimentalis et Applicata* **139**: 215–225.

Mathur V, Tytgat TOG, De Graaf RM, Kalia V, Sankara Reddy A, Vet LEM, Van Dam NM. **2013**. Dealing with double trouble: consequences of single and double herbivory in Brassica juncea. *Chemoecology* **23**: 71–82.

Matsukura K, Matsumura M, Tokuda M. **2012**. Host Feeding by an Herbivore Improves the Performance of Offspring. *Evolutionary Biology* **39**: 341–347.

Mayer RT, Inbar M, McKenzie CL, Shatters R, Borowicz V, Albrecht U, Powell CA, Doostdar H. **2002**. Multitrophic interactions of the silverleaf whitefly, host plants, competing herbivores, and phytopathogens. *Archives of Insect Biochemistry and Physiology* **51**: 151–169.

Mbaluto CM, Ahmad EM, Mädicke A, Grosser K, Van Dam NM, Martínez-Medina A. **2021a**. Induced Local and Systemic Defense Responses in Tomato Underlying Interactions Between the Root-Knot Nematode Meloidogyne incognita and the Potato Aphid Macrosiphum euphorbiae. *Frontiers in Plant Science* **12**: 632212.

Mbaluto CM, Vergara F, Van Dam NM, Martínez-Medina A. **2021b**. Root infection by the nematode *Meloidogyne incognita* modulates leaf antiherbivore defenses and plant resistance to *Spodoptera exigua* (R Hancock, Ed.). *Journal of Experimental Botany* **72**: 7909–7926.

Mcguire RJ, Johnson MTJ. **2006**. Plant genotype and induced responses affect resistance to herbivores on evening primrose (*Oenothera biennis*). *Ecological Entomology* **31**: 20–31.

Megías AG, Müller C. **2010**. Root herbivores and detritivores shape above-ground multitrophic assemblage through plant-mediated effects. *Journal of Animal Ecology* **79**: 923–931.

Mertens D, Fernández de Bobadilla M, Rusman Q, Bloem J, Douma JC, Poelman EH. **2021**. Plant defence to sequential attack is adapted to prevalent herbivores. *Nature Plants* **7**: 1347–1353.

Milano NJ, Barber NA, Adler LS. **2015**. Conspecific and Heterospecific Aboveground Herbivory Both Reduce Preference by a Belowground Herbivore. *Environmental Entomology* **44**: 317–324.

Milbrath LR, Nechols JR. **2004**. Indirect effect of early-season infestations of Trichosirocalus horridus on Rhinocyllus conicus (Coleoptera: Curculionidae). *Biological Control* **30**: 95–109.

Moe SR, Gjørvad IR, Eldegard K, Hegland SJ. **2018**. Ungulate browsing affects subsequent insect feeding on a shared food plant, bilberry (Vaccinium myrtillus). *Basic and Applied Ecology* **31**: 44–51.

Mutua JM, Mutyambai DM, Asudi GO, Khamis F, Niassy S, Jalloh AA, Salifu D, Magara HJO, Calatayud P-A, Subramanian S. **2022**. Competitive Plant-Mediated and Intraguild Predation Interactions of the Invasive Spodoptera frugiperda and Resident Stemborers Busseola fusca and Chilo partellus in Maize Cropping Systems in Kenya. *Insects* **13**: 790.

Nardi C, Rech C, Oliveira JRF, Peñaflor MFGV, Santos F, Bento JMS. **2023**. Preference-performance hypothesis and host oviposition selection of Diabrotica speciosa: aboveground female avoids belowground conspecific larvae in maize. *Arthropod-Plant Interactions* **17**: 629–638.

Nazeri M, Allahyari H, Goldansaz H. **2018**. Reciprocal indirect interactions between Tetranychus urticae and Aphis gossypii mediated by cucumber plant. *Journal of Asia-Pacific Entomology* **21**: 843–851.

Nombela G, Garzo E, Duque M, Muñiz M. **2009**. Preinfestations of tomato plants by whiteflies (*Bemisia tabaci*) or aphids (*Macrosiphum euphorbiae*) induce variable resistance or susceptibility responses. *Bulletin of Entomological Research* **99**: 183–191.

Nordkvist M, Klapwijk MJ, Edenius L, Gershenzon J, Schmidt A, Björkman C. **2019**. Trait-mediated indirect interactions: Moose browsing increases sawfly fecundity through plant-induced responses. *Ecology and Evolution* **9**: 10615–10629.

Ntiri ES, Calatayud P-A, Musyoka B, Van Den Berg J, Le Ru BP. **2018**. Influence of feeding-damaged plants on the oviposition responses within a community of female moths. *Phytoparasitica* **46**: 607–615.

Peñaflor MFGV, Andrade FM, Sales L, Silveira EC, Santa-Cecília LVC. **2019**. Interactions between white mealybugs and red spider mites sequentially colonizing coffee plants. *Journal of Applied Entomology* **143**: 957–963.

Pereira RV, Filgueiras CC, Willett DS, Peñaflor MFGV. **2020**. Sight unseen: Belowground feeding influences the distribution of an aboveground herbivore. *Ecosphere* **11**: e03163.

Pérez-Hedo M, Arias-Sanguino ÁM, Urbaneja A. **2018**. Induced Tomato Plant Resistance Against Tetranychus urticae Triggered by the Phytophagy of Nesidiocoris tenuis. *Frontiers in Plant Science* **9**: 1419.

Pierre PS, Dugravot S, Cortesero A-M, Poinsot D, Raaijmakers CE, Hassan HM, Van Dam NM. **2012**. Broccoli and turnip plants display contrasting responses to belowground induction by Delia radicum infestation and phytohormone applications. *Phytochemistry* **73**: 42–50.

Pineda A, Soler R, Pastor V, Li Y, Dicke M. **2017**. Plant-mediated species networks: the modulating role of herbivore density. *Ecological Entomology* **42**: 449–457.

Pingault L, Basu S, Zogli P, Williams WP, Palmer N, Sarath G, Louis J. **2021**. Aboveground Herbivory Influences Belowground Defense Responses in Maize. *Frontiers in Ecology and Evolution* **9**: 765940.

Piubelli GC, Hoffmann-Campo CB, Arruda ICD. **2003**. Flavonoid increase in soybean as a response to Nezara viridula injury and its effect on insect feeding preference. *Journal of Chemical Ecology*.

Poelman EH, Broekgaarden C, Van Loon JJA, Dicke M. **2008**. Early season herbivore differentially affects plant defence responses to subsequently colonizing herbivores and their abundance in the field. *Molecular Ecology* **17**: 3352–3365.

Poelman EH, Zheng S-J, Zhang Z, Heemskerk NM, Cortesero A-M, Dicke M. **2011**. Parasitoid-specific induction of plant responses to parasitized herbivores affects colonization by subsequent herbivores. *Proceedings of the National Academy of Sciences* **108**: 19647–19652.

Puri H, Ikuze E, Ayala J, Rodriguez I, Kariyat R, Louis J, Grover S. **2023**. Greenbug feeding-induced resistance to sugarcane aphids in sorghum. *Frontiers in Ecology and Evolution* **11**: 1105725.

Quintero C, Bowers MD. **2011**. Plant Induced Defenses Depend More on Plant Age than Previous History of Damage: Implications for Plant-Herbivore Interactions. *Journal of Chemical Ecology* **37**: 992–1001.

Ramirez RA, Spears LR. **2014**. Stem Nematode Counteracts Plant Resistance of Aphids in Alfalfa, Medicago Sativa. *Journal of Chemical Ecology* **40**: 1099–1109.

Ray S, Helms AM, Matulis NL, Davidson-Lowe E, Grisales W, Ali JG. **2020**. Asymmetry in Herbivore Effector Responses: Caterpillar Frass Effectors Reduce Performance of a Subsequent Herbivore. *Journal of Chemical Ecology* **46**: 76–83.

Rechner O, Laurenz S, Hondelmann P, Poehling H-M. **2017**. Local and systemic interactions of Aulacorthum solani and Myzus persicae on tomato. *Journal of Plant Diseases and Protection* **124**: 289–294.

Rieske LK, Raffa KF. **1998**. Interactions Among Insect Herbivore Guilds: Influence of Thrips Bud Injury on Foliar Chemistry and Suitability to Gypsy Moths.

Rigsby CM, Shoemaker EE, Mallinger MM, Orians CM, Preisser EL. **2019**. Conifer responses to a stylet-feeding invasive herbivore and induction with methyl jasmonate: impact on the expression of induced defences and a native folivore. *Agricultural and Forest Entomology* **21**: 227–234.

Rim H, Uefune M, Ozawa R, Takabayashi J. **2018**. An omnivorous arthropod, Nesidiocoris tenuis, induces gender-specific plant volatiles to which conspecific males and females respond differently. *Arthropod-Plant Interactions* **12**: 495–503.

Ripa L, Stevens GN, Lewis EE. **2023**. Two-way plant-mediated interactions between a plant parasitic nematode and a foliar herbivore arthropod. *Rhizosphere* **26**: 100699.

Robert CAM, Erb M, Duployer M, Zwahlen C, Doyen GR, Turlings TCJ. **2012a**. Herbivore-induced plant volatiles mediate host selection by a root herbivore. *New Phytologist* **194**: 1061–1069.

Robert CAM, Erb M, Hibbard BE, Wade French B, Zwahlen C, Turlings TCJ. **2012b**. A specialist root herbivore reduces plant resistance and uses an induced plant volatile to aggregate in a density-dependent manner (K Thompson, Ed.). *Functional Ecology* **26**: 1429–1440.

Rodriguez-Saona C, Chalmers JA, Raj S, Thaler JS. **2005**. Induced plant responses to multiple damagers: differential effects on an herbivore and its parasitoid. *Oecologia* **143**: 566–577.

Schott J, Jantzen F, Hilker M. **2023**. Elm tree defences against a specialist herbivore are moderately primed by an infestation in the previous season (P Bonello, Ed.). *Tree Physiology* **43**: 1218–1232.

Shi J-H, Liu H, Pham TC, Hu X-J, Liu L, Wang C, Foba CN, Wang S-B, Wang M-Q. **2022**. Volatiles and hormones mediated root-knot nematode induced wheat defense response to foliar herbivore aphid. *Science of The Total Environment* **815**: 152840.

Shivaramu S, Jayanthi PDK, Kempraj V, Anjinappa R, Nandagopal B, Chakravarty AK. **2017**. What signals do herbivore-induced plant volatiles provide conspecific herbivores? *Arthropod-Plant Interactions* **11**: 815–823.

Silva DB, Jiménez A, Urbaneja A, Pérez-Hedo M, Bento JM. **2021**. Changes in plant responses induced by an arthropod influence the colonization behavior of a subsequent herbivore. *Pest Management Science* **77**: 4168–4180.

Silva R, Walter GH, Wilson LJ, Furlong MJ. **2014**. Responses of *T hrips tabaci* to odours of herbivore-induced cotton seedlings. *Entomologia Experimentalis et Applicata* **151**: 239–246.

Silva R, Walter GH, Wilson LJ, Furlong MJ. **2017**. Effects of single and dual species herbivory on the behavioral responses of three thrips species to cotton seedlings. *Insect Science* **24**: 684–698.

Simelane DO. **2006**. Effect of herbivory by Teleonemia scrupulosa on the performance of Longitarsus bethae on their shared host, Lantana camara. *Biological Control* **39**: 385–391.

Soler R, Badenes-pérez FR, Broekgaarden C, David A, Boland W, Dicke M, Soler R, Badenes-pérez FR, Broekgaarden C, David A, *et al.* **2012**. Plant-mediated facilitation between a leaf-feeding and a phloem-feeding insect in a brassicaceous plant: from insect performance to gene transcription. *Functional Ecology* **26**: 156–166.

Soler R, Bezemer TM, Cortesero AM, Van Der Putten WH, Vet LEM, Harvey JA. **2007**. Impact of foliar herbivory on the development of a root-feeding insect and its parasitoid. *Oecologia* **152**: 257–264.

Soler R, Harvey JA, Rouchet R, Schaper SV, Martijn Bezemer T. **2010**. Impacts of belowground herbivory on oviposition decisions in two congeneric butterfly species: Above-belowground plant-insect interactions. *Entomologia Experimentalis et Applicata* **136**: 191–198.

Soler R, Schaper SV, Bezemer TM, Cortesero AM, Hoffmeister TS, Van Der Putten WH, Vet LEM, Harvey JA. **2009**. Influence of presence and spatial arrangement of belowground insects on host-plant selection of aboveground insects: A field study. *Ecological Entomology* **34**: 339–345.

Sousa ALV, Silva DB, Silva GG, Bento JMS, Penãflor MFGV, Souza B. **2020**. Behavioral response of the generalist predator Orius insidiosus to single and multiple herbivory by two cell content-feeding herbivores on rose plants. *Arthropod-Plant Interactions* **14**: 227–236.

Staley JT, Mortimer SR, Morecroft MD, Brown VK, Masters GJ. **2007**. Summer drought alters plant-mediated competition between foliar- and root-feeding insects. *Global Change Biology* **13**: 866–877.

Stam JM, Chrétien L, Dicke M, Poelman EH. **2017**. Response of Brassica oleracea to temporal variation in attack by two herbivores affects preference and performance of a third herbivore. *Ecological Entomology* **42**: 803–815.

Su Q, Chen G, Mescher MC, Peng Z, Xie W, Wang S, Wu Q, Liu J, Li C, Wang W, *et al.* **2018**. Whitefly aggregation on tomato is mediated by feeding-induced changes in plant metabolites that influence the behaviour and performance of conspecifics (E Gianoli, Ed.). *Functional Ecology* **32**: 1180–1193.

Sun X, Siemann E, Liu Z, Wang Q, Wang D, Huang W, Zhang C, Ding J. **2019**. Root-feeding larvae increase their performance by inducing leaf volatiles that attract above-ground conspecific adults (T Züst, Ed.). *Journal of Ecology* **107**: 2713–2723.

Sun X, Sun Y, Ma L, Liu Z, Zhang C, Huang W, Siemann E, Ding J. **2022**. Linking aboveground and belowground interactions via herbivore-induced plant volatiles. *Entomologia Generalis* **42**: 421–429.

Tabuchi K, Quiring DT, Flaherty LE, Pinault LL, Ozaki K. **2011**. Bottom-up trophic cascades caused by moose browsing on a natural enemy of a galling insect on balsam fir. *Basic and Applied Ecology* **12**: 523–531.

Tabuchi K, Ueda A, Ozaki K. **2010**. Contrasting effects of deer browsing on oviposition preference, neonate survival and potential fecundity of a galling insect. *Écoscience* **17**: 379–386.

Thompson T, Bidart MG. **2017**. Oviposition Preferences of Plutella xylostella are Influenced by the Type of Plant Induction and Glucosinolate Hydrolysis Profiles. *Journal of Insect Behavior* **30**: 507–518.

Thompson MN, Grunseich JM, Marmolejo LO, Aguirre NM, Bradicich PA, Behmer ST, Suh CP-C, Helms AM. **2022**. Undercover operation: Belowground insect herbivory modifies systemic plant defense and repels aboveground foraging insect herbivores. *Frontiers in Ecology and Evolution* **10**: 1033730.

Tian T, Zhi J-R, Lv Z-Y, Wen J, Cao Y. **2017**. Preference of *Tetranychus urticae* (Acari: Tetranychidae) for Kidney Beans Pre-Infested by *Frankliniella occidentalis* (Thysanoptera: Thripidae) and Possible Roles of Induced Bean Volatiles. *Journal of the Kansas Entomological Society* **90**: 313–322.

Tindall KV, Stout MJ. **2001**. Plant-mediated interactions between the rice water weevil and fall armyworm in rice. *Entomologia Experimentalis et Applicata* **101**: 9–17.

Underwood N. **2012**. When herbivores come back: effects of repeated damage on induced resistance (S Carroll, Ed.). *Functional Ecology* **26**: 1441–1449.

Vaello T, Pineda A, Marcos-García MÁ. **2019**. Role of Thrips Omnivory and Their Aggregation Pheromone on Multitrophic Interactions Between Sweet Pepper Plants, Aphids, and Hoverflies. *Frontiers in Ecology and Evolution* **6**: 240.

Vaello T, Sarde SJ, Marcos-García MÁ, De Boer JG, Pineda A. **2018**. Modulation of plant-mediated interactions between herbivores of different feeding guilds: Effects of parasitism and belowground interactions. *Scientific Reports* **8**: 14424.

Valladares GA, Coll-Aráoz MV, Alderete M, Vera MT, Fernández PC. **2020**. Previous herbivory alerts conspecific gravid sawflies to avoid unsuitable host plants. *Bulletin of Entomological Research* **110**: 438–448.

Van Dam NM, Raaijmakers CE, Van Der Putten WH. **2005**. Root herbivory reduces growth and survival of the shoot feeding specialist *Pieris rapae* on *Brassica nigra*. *Entomologia Experimentalis et Applicata* **115**: 161–170.

Van Dam NM, Wondafrash M, Mathur V, Tytgat TOG. **2018**. Differences in Hormonal Signaling Triggered by Two Root-Feeding Nematode Species Result in Contrasting Effects on Aphid Population Growth. *Frontiers in Ecology and Evolution* **6**: 88.

Van Geem M, Gols R, Raaijmakers CE, Harvey JA. **2016**. Effects of population-related variation in plant primary and secondary metabolites on aboveground and belowground multitrophic interactions. *Chemoecology* **26**: 219–233.

Van Zandt PA, Agrawal AA. **2004**. Specificity of induced plant responses to specialist herbivores of the common milkweed *Asclepias syriaca*. *Oikos* **104**: 401–409.

Vandegehuchte ML, De La Peña E, Bonte D. **2010**. Interactions between root and shoot herbivores of Ammophila arenaria in the laboratory do not translate into correlated abundances in the field. *Oikos* **119**: 1011–1019.

Varsani S, Basu S, Williams WP, Felton GW, Luthe DS, Louis J. **2016**. Intraplant communication in maize contributes to defense against insects. *Plant Signaling & Behavior* **11**: e1212800.

Viswanathan DV, Lifchits OA, S. TJ. **2007**. Consequences of Sequential Attack for Resistance to Herbivores When Plants Have Specific Induced Responses. *Oikos* **116**: 1389–1399.

Wan J, Yi J, Tao Z, Ren Z, Otieno EO, Tian B, Ding J, Siemann E, Erb M, Huang W. **2022**. Species-specific plant-mediated effects between herbivores converge at high damage intensity. *Ecology* **103**: e3647.

Wang M, Bezemer TM, Van Der Putten WH, Biere A. **2015**. Effects of the Timing of Herbivory on Plant Defense Induction and Insect Performance in Ribwort Plantain (Plantago lanceolata L.) Depend on Plant Mycorrhizal Status. *Journal of Chemical Ecology* **41**: 1006–1017.

Wang M, Biere A, Van Der Putten WH, Bezemer TM. **2014**. Sequential effects of root and foliar herbivory on aboveground and belowground induced plant defense responses and insect performance. *Oecologia* **175**: 187–198.

Weeraddana CDS, Evenden ML. **2019**. Herbivore-induced plants do not affect oviposition but do affect fitness of subsequent herbivores on canola. *Entomologia Experimentalis et Applicata* **167**: 341–349.

Wei H, He M, Lu X, Ding J. **2016**. Differences in interactions of aboveground and belowground herbivores on the invasive plant Alternanthera philoxeroides and native host A. sessilis. *Biological Invasions* **18**: 3437–3447.

Wurst S, Van Der Putten WH. **2007**. Root herbivore identity matters in plant-mediated interactions between root and shoot herbivores. *Basic and Applied Ecology* **8**: 491–499.

Xu T. **2002**. Effects of herbivore-induced rice volatiles on the host selec-tion behavior of brown planthopper, Nilaparvata lugens. *Chinese Science Bulletin* **47**: 1355.

Yang S, Guo W, Tang J, Wang W, Wu Q, Li X. **2022**. Impact of caterpillars and plant-parasitic nematodes on Chinese tallow tree are more strongly affected by nematode density than by caterpillar identity. *Arthropod-Plant Interactions* **16**: 119–128.

Zhang Y, Fan J, Fu Y, Francis F, Chen J. **2019**. Plant-Mediated Interactions between Two Cereal Aphid Species: Promotion of Aphid Performance and Attraction of More Parasitoids by Infestation of Wheat with Phytotoxic Aphid *Schizaphis graminum*. *Journal of Agricultural and Food Chemistry* **67**: 2763–2773.

Zhao H, Zhang X, Xue M, Zhang X. **2015**. Feeding of Whitefly on Tobacco Decreases Aphid Performance via Increased Salicylate Signaling (X-W Wang, Ed.). *PLOS ONE* **10**: e0138584.

Zhou Y, Giusti MM, Parker J, Salamanca J, Rodriguez-Saona C. **2016**. Frugivory by Brown Marmorated Stink Bug (Hemiptera: Pentatomidae) Alters Blueberry Fruit Chemistry and Preference by Conspecifics. *Environmental Entomology* **45**: 1227–1234.
